# A Benchmark and Basis-Set Extrapolation Study of Hyperfine Coupling Constants from the Random Phase Approximation and σ‑Functionals

**DOI:** 10.1021/acs.jpca.6c00623

**Published:** 2026-04-23

**Authors:** Daniel Graf, Lu Liu, Florian Siekmann, Viktoria Drontschenko, Christian Ochsenfeld

**Affiliations:** † Theoretical Chemistry, Department of Chemistry, Ludwig-Maximilians-Universität München (LMU), D-81377 Munich, Germany; ‡ Max Planck Institute for Solid State Research, D-70569 Stuttgart, Germany

## Abstract

Hyperfine coupling constants (HFCCs) provide an important link between theory and experiment; however, their accurate computation at a reasonable computational cost remains challenging. In this work, we present the theory for evaluating HFCCs on the level of the promising σ-functionals developed in the Görling group. We show that the best-performing σ-functional achieves accuracies, relative to coupled-cluster singles doubles and perturbative triples (CCSD­(T)) reference values, comparable to those of domain-based local pair-natural orbital coupled-cluster singles doubles (DLPNO–CCSD)—currently among the most accurate methods applicable beyond the few-atoms scale—while being significantly less computationally demanding. We further investigate the influence of the atomic-orbital basis on random phase approximation (RPA), σ-functional, and also CCSD­(T) HFCCs, showing that the pcH and pcJ basis-set families developed in the Jensen group are well suited for these methods. The availability of these basis-set families enables systematic complete basis-set (CBS) extrapolations for all HFCC calculations, with different extrapolation schemes producing very similar results. The presented basis-set extrapolated CCSD­(T) HFCCs can hence serve as new reference values for future method developments or benchmarks.

## Graphical abstract

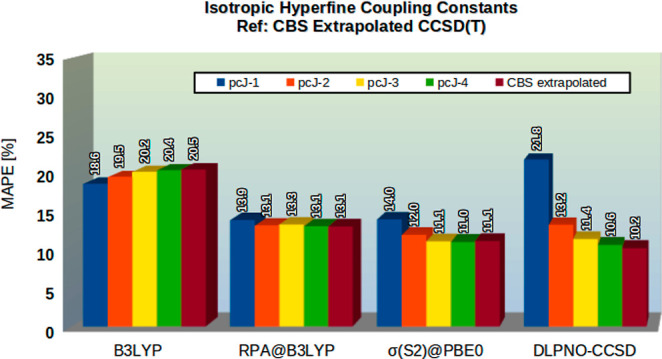

## 1 Introduction

Electron spin resonance (ESR), also known as electron paramagnetic resonance (EPR), is the electronic analog of nuclear magnetic resonance (NMR). In EPR spectroscopy, the g-tensor and the electron–nucleus hyperfine coupling constants (HFCCs) are among the most important parameters. The g-tensor—the analog of the nuclear magnetic shielding tensor in NMR—describes the interaction of the electron spin with an external magnetic field, whereas the HFCCs—the analogs of the J-couplings in NMR—capture the interaction between the electron spin and the various nuclear magnetic moments of the system. Together, these quantities provide insight into the spin-density distribution within the molecule under study.^1^

In analogy with NMR, the g-tensor and the HFCCs are sensitive to the molecular environment and hence carry structural information which can be used for deducing the molecular structure. Owing to these characteristics, EPR has become an indispensable tool to characterize paramagnetic species such as organic radicals or transition-metal catalysts.^1^

Hyperfine coupling constants can be decomposed into isotropic (Fermi-contact, FC), anisotropic (spin-dipolar, SD), and spin–orbit (SO) contributions. For rapidly moving molecules in the gas phase or in solution, the SD and the anisotropic part of the SO contribution are motionally averaged to zero, so only the isotropic hyperfine coupling is reflected in the observed EPR hyperfine structure. Nevertheless, the anisotropic contributions remain relevant as their fluctuations contribute to relaxation processes and become directly observable in the solid state.^2^ The SO contribution is usually small for light nuclei common in organic radicals and can therefore be omitted;^1^ however, for heavier elements, particularly in transition-metal complexes, both spin–orbit and scalar relativistic effects become increasingly important. More specifically, relativistic effects on HFCCs can be substantial for systems containing atoms heavier than Xe.^1^ Consistent with this, a recent study showed that for first- and second-row elements, the relativistic treatments DKH2 (second-order Douglas–Kroll–Hess) and X2C (exact two-component) yield negligible differences.^3^

To support experiments by validating, interpreting, and complementing results, quantum chemical calculations are of great importance; nevertheless, the accurate computation of magnetic properties remains challenging. Although sophisticated wave function approaches like coupled cluster (CC) or multiconfigurational self-consistent field (CASSCF) methods are preferred for highly accurate predictions of EPR parameters,^4,5^ their very high computational cost restricts their application to small systems comprising only a few atoms. For larger systems, density functional theory (DFT) is typically the approach of choice (and often the only feasible option) due to its excellent cost-to-performance ratio. Unfortunately, the accuracy achievable with today’s density functionals is often markedly lower than that of CC methods and can vary substantially depending on the system under study.^5–8^ To address this issue, an efficient coupled-cluster approach—the domain-based local pair-natural orbital CC singles doubles (DLPNO–CCSD) method—has been proposed,^9^ opening the door to coupled-cluster HFCC calculations on large molecular systems.

It is well established that the CCSD method can provide high accuracy for the calculation of HFCCs^7,10,11^ and also its approximate DLPNO variant has been shown to improve significantly over density functional approximations (DFAs).^6,9,12^ However, even with very tight thresholds and for relatively small systems, the DLPNO approximation can introduce notable deviations from canonical CCSD.^9^ Moreover, due to possible changes in correlation domains with variations in the molecular geometry—relevant, e.g., in vibrational perturbation theory for evaluating vibrational corrections—discontinuities may arise in the HFCC surfaces obtained with DLPNO–CCSD,^12^ potentially resulting in numerical instabilities. Finally, although significantly more efficient than canonical CCSD, DLPNO–CCSD still comes with a substantial computational cost.

A theoretical framework that could offer a viable alternative to the DLPNO–CCSD method is the random phase approximation^13–16^ (RPA) and its extension to the closely related σ-functionals.^17–22^ The probably most popular derivation of the RPA is the one presented by Langreth and Perdew dating back to 1975,^23^ which makes use of an adiabatic connection between the noninteracting Kohn–Sham^24^ and the fully interacting system together with the fluctuation–dissipation theorem,^25^ allowing for an exact expression of the exchange–correlation energy in terms of density–density response functions known from time-dependent density functional theory^26^ (TDDFT). Alternatively, derivations within the realm of wave function theory also exist, demonstrating the equivalence of the (direct) RPA and ring coupled-cluster doubles.^27^

In its original formulation, the RPA scales with $O\left(M^{6}\right)$ where *M* is a measure of the system size,^28^ but the scaling can be reduced to $O\left(M^{4}\right)$ using a resolution-of-the-identity (RI) approach^29^ and further to O(M) if the locality of the electronic structure is exploited.^30–33^ Building on the RPA framework, σ-functionals introduce an additional empirical correction term, which is parametrized by optimizing reaction, transition-state, atomization, and noncovalent interaction energies from established benchmark sets.^17^ Although these σ-functionals offer substantially improved accuracy,^34–38^ their computational cost remains comparable to that of standard RPA, rendering them highly attractive.

In a recent HFCC study by Bruder et al.,^39^ RPA was shown to outperform global, range-separated, and local hybrid functionals for main-group systems, achieving accuracies close to CCSD while maintaining significantly lower computational cost; similar findings have been reported for NMR shieldings.^34,40,41^ Moreover, the most recent σ-functionals were found to yield even higher accuracy than RPA for NMR shieldings,^34,41^ suggesting that comparable performance may be achievable for HFCCs as well.

Motivated by these findings, we here present the theoretical framework for calculating nonrelativistic HFCCs at the level of σ-functionals (Section 2). We begin by deriving an expression for the HFCCs from a general energy formulation. We then consider the specific energy expression for the σ-functionals and derive the corresponding expression for HFCCs by analytically differentiating the energy with respect to the nuclear magnetic moment of a given nucleus. Computational details are provided in Section 3. In Section 4, we assess the accuracy of RPA and several σ-functionals for two test sets consisting of small and large main-group compounds originally introduced by the Bartlett group.^5^ Starting with the small-molecule test set, we first establish new complete-basis-set extrapolated CCSD­(T) reference values utilizing specialized basis sets developed in the Jensen group.^2^ We then investigate and discuss the accuracy of RPA based on different input orbitals as well as several σ-functionals. The influence of the auxiliary basis on both the RPA and σ-functional results is subsequently examined, before extending the discussion to the set of larger main-group compounds. Finally, our concluding remarks are provided in Section 5.

## 2 Theory

### 2.1 Notation

The notation used in this work is summarized in Table 1. For two- and three-center integrals the Mulliken notation is employed. For general intermediates containing only integrals, the derivative with respect to a general perturbation ξ is denoted as *O* ^(ξ)^. In all other cases, *O* ^ξ^ is used as a short-hand notation for $\frac{\partial }{\partial \xi }O$ .

**Table 1:** Notation Used in This Work

symbols	meaning
μ, ν, λ, σ	atomic orbital indices
*M*, *N*, *O*, *P*, *Q*	auxiliary function indices
*i*, *j*	electron indices
*u*, *v*	Cartesian coordinates (*x*, *y*, *z*)
*N*_AO_	total number of AO basis functions
*N*_aux_	total number of auxiliary functions
*N*_el_	total number of electrons

### 2.2 Computation of Hyperfine Coupling Constants

The hyperfine coupling constant (HFCC) of a nucleus *A* is proportional to the first derivative of the total energy with respect to the nuclear magnetic moment **m**_*A*_, evaluated at zero perturbation. For a density-matrix-based energy expression, this derivative involves the partial derivatives of the core Hamiltonian matrix **h** and the one-particle density matrix **P** ^42,43^

1 $$\frac{\partial E}{\partial m_{A}}=Tr\left(\frac{\partial E}{\partial h}\frac{\partial h}{\partial m_{A}}\right)+Tr\left(\frac{\partial E}{\partial P}\frac{\partial P}{\partial m_{A}}\right)$$

Because the σ-functional framework employs a nonvariational reference density, the derivative of the density matrix with respect to the perturbation (**P** ^**m**_*A*_^) must be accounted for. However, the explicit calculation of **P** ^**m**_*A*_^ can be bypassed using the Z-vector technique of Handy and Schaefer.^44,45^ By solving a single coupled-perturbed self-consistent-field (CPSCF) equation, the orbital relaxation contributions can be absorbed into an effective relaxed density matrix **Q**. Since the molecular basis functions are independent of **m**_*A*_, the energy derivative simplifies to the trace over the perturbed core Hamiltonian

2 $$\frac{\partial E}{\partial m_{A}}=Tr\left(Qh^{m_{A}}\right)$$

Neglecting spin–orbit coupling, the derivative of the core Hamiltonian with respect to **m**_*A*_ reduces to the Fermi contact (FC) and spin-dipole (SD) operators.^1^ Consequently, eq 2 can be split into isotropic and anisotropic components

3 $$\frac{\partial E}{\partial m_{A}}=\underset{≔A_{A}^{FC}}{\underset{︸}{Tr\left(Qh_{A}^{FC}\right)}}+\underset{≔A_{A,uv}^{SD}}{\underset{︸}{Tr\left(Qh_{A}^{SD}\right)}}$$

Evaluating these traces in atomic units yields the working equations for the FC and SD contributions to the HFCC^39,46,47^

4 $$A_{A}^{FC}=\frac{4\pi }{3c^{2}}\frac{2}{N_{\alpha }-N_{\beta }}\beta_{e}g_{e}\beta_{A}g_{A}\sum_{\mu \nu }^{N_{AO}}Q_{\mu \nu }\langle \mu |\delta \left(r_{A}\right)|\nu \rangle$$

5 $$A_{A,uv}^{SD}=\frac{1}{2c^{2}}\frac{2}{N_{\alpha }-N_{\beta }}\beta_{e}g_{e}\beta_{A}g_{A}\sum_{\mu \nu }^{N_{AO}}Q_{\mu \nu }\langle \mu |\frac{3r_{A,u}r_{A,v}-\delta_{uv}r_{A}^{2}}{r_{A}^{5}}|\nu \rangle$$

where *N*_α_ and *N*_β_ are the numbers of α and β electrons, respectively; **r**_*A*_ is the electron–nucleus position vector with norm *r*_*A*_; β_e_ and β_*A*_ are the Bohr and nuclear magnetons; *g*_e_ and *g*_*A*_ are the electron and nuclear *g*-factors; δ­(**r**_*A*_) is the Dirac delta function; δ_*uv*_ is the Kronecker delta; and *c* is the speed of light in a vacuum.

### 2.3 σ-Functional Hyperfine Coupling Constants

#### 2.3.1 RPA and σ-Functional Energy Expressions

The total energy *E*_total_ within the adiabatic-connection fluctuation–dissipation theorem^23,48,49^ is given by

6 $$E_{total}=E_{HF}\left[P\right]+E_{c}\left[P\right]$$

where *E*_HF_[**P**] and *E*_c_[**P**] denote the Hartree–Fock (HF) and correlation energy functional, respectively, both evaluated using the one-particle density matrix, **P**, stemming from a prior HF or DFT calculation. The correlation energy within the σ-functional formalism can be expressed as^17,18^

7 $$E_{c}^{\sigma }=-\frac{1}{2\pi }\int_{0}^{\infty }d\omega Tr\left(\underset{≔H^{RPA}\left(\sigma \left(i\omega \right)\right)}{\underset{︸}{-\ln\left(1+\sigma \left(i\omega \right)\right)+\sigma \left(i\omega \right)}}+H^{\sigma }\left(\sigma \left(i\omega \right)\right)\right)$$

where **H** ^σ^(**σ**(iω)) (defined in Appendix A) is an additional term that is added to the standard RPA expression, optimized using energetic reference data to correct the RPA error arising from neglecting the exchange–correlation kernel. Further, −**σ**(iω) is the frequency dependent eigenvalue matrix of the noninteracting response function **X**_0_(iω) multiplied by the electron–electron interaction operator in the auxiliary basis **C**, which is given by

8 $$C_{MN}=\sum_{PQ}^{N_{aux}}{\left(M\left|m_{12}\right|P\right)}^{-1}\left(P|Q\right){\left(Q\left|m_{12}\right|N\right)}^{-1}$$

where *m*_12_ denotes a general RI metric. A symmetric expression for **X**_0_(iω)**C**, containing the same eigenvalues, can be obtained, e.g., via a symmetric decomposition as follows

9 $$-C^{1/2}X_{0}\left(i\omega \right)C^{1/2}=V\left(i\omega \right)\sigma \left(i\omega \right)V^{T}\left(i\omega \right)$$

10 $$\sigma \left(i\omega \right)=-V^{T}\left(i\omega \right)\left[C^{1/2}X_{0}\left(i\omega \right)C^{1/2}\right]V\left(i\omega \right)$$

with the eigenvector matrix **V**(iω). Another possible decomposition is the Cholesky decomposition, defined as **C** = **LL** ^T^. In the following, however, we employ the symmetric expression of eq 10, which can be readily adapted to the Cholesky-decomposed form.

#### 2.3.2 HFCCs

Taking the derivative of the total energy given in eq 6 with respect to the nuclear magnetic moment of a nucleus *A* yields

11 $$E_{total}^{\sigma ,m_{A}}=E_{HF}^{m_{A}}\left[P\right]+E_{c}^{\sigma ,m_{A}}\left[P\right]$$

12 $$=Tr\left(h^{m_{A}}P\right)+Tr\left(H_{HF}P^{m_{A}}\right)+E_{c}^{\sigma ,m_{A}}\left[P\right]$$

where **H**_HF_ represents the Fock matrix. The derivative of the σ-functional correlation energy can be expressed as

13 $$E_{c}^{\sigma ,m_{A}}=-\frac{1}{2\pi }\int_{0}^{\infty }d\omega Tr\left(\frac{\partial E_{c}^{\sigma }\left(i\omega \right)}{\partial \sigma \left(i\omega \right)}\frac{\partial \sigma \left(i\omega \right)}{\partial m_{A}}\right)$$

The first term in the trace can be obtained by differentiating eq 7 with respect to **σ**

14 $$\frac{\partial E_{c}^{\sigma }\left(i\omega \right)}{\partial \sigma \left(i\omega \right)}=-\frac{\partial \left[\ln\left(1+\sigma \left(i\omega \right)\right)\right]}{\partial \sigma \left(i\omega \right)}+1+\frac{\partial H^{\sigma }\left(\sigma \left(i\omega \right)\right)}{\partial \sigma \left(i\omega \right)}$$

15 $$=-{\left[\sigma \left(i\omega \right)+1\right]}^{-1}+1+\frac{\partial H^{\sigma }\left(\sigma \left(i\omega \right)\right)}{\partial \sigma \left(i\omega \right)}$$

The last term, i.e. $\frac{\partial H^{\sigma }\left(\sigma \left(i\omega \right)\right)}{\partial \sigma \left(i\omega \right)}$ , is straightforward as it only requires differentiation of a polynomial expression. Its explicit form is given in Appendix A.

Next, we derive an expression for $\frac{\partial \sigma \left(i\omega \right)}{\partial m_{A}}$ , the last remaining term required for computing σ-functional HFCCs. Using eq 10, the partial derivative of σ can be expressed as

16 $$\frac{\partial \sigma }{\partial m_{A}}=-\frac{\partial \left(V^{T}\left(i\omega \right)\left[C^{1/2}X_{0}\left(i\omega \right)C^{1/2}\right]V\left(i\omega \right)\right)}{\partial m_{A}}$$

Since the eigenvector matrix **V** is orthonormal and the matrix **C** is independent of the nuclear magnetic moment, the above equation simplifies to

17 $$\frac{\partial \sigma }{\partial m_{A}}=-V^{T}\left(i\omega \right)C^{1/2}\frac{\partial X_{0}\left(i\omega \right)}{\partial m_{A}}C^{1/2}V\left(i\omega \right)$$

Please note that in the above and subsequent equations, the trace implies integration over imaginary frequencies wherever applicable. Inserting eqs 17 into [Disp-formula eq13] and using the resulting expression in eq 12 gives

18 $$\begin{array}{ll} E_{total}^{\sigma ,m_{A}} & =Tr\left(h^{m_{A}}P\right)+Tr\left(H_{HF}P^{m_{A}}\right)\\ & -Tr\left(\frac{\partial E_{c}^{\sigma }}{\partial \sigma \left(i\omega \right)}V^{T}\left(i\omega \right)C^{1/2}\frac{\partial X_{0}\left(i\omega \right)}{\partial m_{A}}C^{1/2}V\left(i\omega \right)\right)\\ & =Tr\left(h^{m_{A}}P\right)+Tr\left(H_{HF}P^{m_{A}}\right)+Tr\left(W_{c}^{\sigma }\left(i\omega \right)\frac{\partial X_{0}\left(i\omega \right)}{\partial m_{A}}\right) \end{array}$$

with

19 $$W_{c}^{\sigma }\left(i\omega \right)=-C^{1/2}V\left(i\omega \right)\frac{\partial E_{c}^{\sigma }}{\partial \sigma \left(i\omega \right)}V^{T}\left(i\omega \right)C^{1/2}$$

Considering the partial derivative of the noninteracting response function with respect to the density matrix yields

20 $$E_{total}^{m_{A}}=Tr\left(h^{m_{A}}P\right)+Tr\left(H_{HF}P^{m_{A}}\right)+Tr\left(W_{c}^{\sigma }\left(i\omega \right)\frac{\partial X_{0}\left(i\omega \right)}{\partial P}\frac{\partial P}{\partial m_{A}}\right)$$

As outlined in Section 2.2, the explicit evaluation of the perturbed density matrix in eq 20 is avoided by employing the Z-vector method. Specifically, the factors multiplying the perturbed density matrix in the last two terms of eq 20 represent the partial derivative of the energy with respect to the unperturbed density matrix. By using this derivative to solve the associated coupled-perturbed equations, the orbital relaxation effects are absorbed entirely into the effective relaxed density matrix **Q**. This reduces the total energy derivative to the compact form of eq 2, which requires only the trace with the perturbation-dependent core Hamiltonian **h** ^**m**_*A*_^, thereby providing direct access to the FC and SD contributions.

#### 2.3.3 Comparison to the RPA HFCC Expression

Using the RPA gradient expression of ref 50, the derivative of the RPA energy with respect to the nuclear magnetic moment can be expressed as

21 $$E_{total}^{m_{A}}=Tr\left(h^{m_{A}}P\right)+Tr\left(H_{HF}P^{m_{A}}\right)+Tr\left(W_{c}\left(i\omega \right)\frac{\partial X_{0}\left(i\omega \right)}{\partial P}\frac{\partial P}{\partial m_{A}}\right)$$

where

22 $$W_{c}\left(i\omega \right)=-4\pi \frac{\partial E_{c}^{RPA}}{\partial X_{0}\left(i\omega \right)}$$

represents the correlated screened-Coulomb interaction in the auxiliary basis^50,51^ —a central quantity in many-body perturbation theory, widely known from *GW* theory.^51–53^

A comparison of the σ-functional (eq. 20) and the RPA (eq. 21) expressions reveals that the only difference lies in the screened-Coulomb interaction. This demonstrates that an existing RPA implementation can be seamlessly upgraded to the σ-functional level by simply replacing the standard RPA screened-Coulomb interaction **W**_c_(iω) with the modified σ-functional interaction **W**_c_^σ^(iω) when evaluating the correlation energy derivative for the Z-vector equations.

## 3 Computational Details

The two sets of radicals used in this work, covering small and large main-group compounds, were taken from ref 5 of the Bartlett group. Structures were adopted from the literature along with the available experimental results.^5^ The first test set consists of 26 molecules with fewer than 6 atoms and comprises 65 distinct isotropic HFCCs, while the second test set comprises 8 larger radicals and in total 73 isotropic HFCC values. The geometries of all compounds used in this work can be found in the Supporting Information. Zn-porphycene from the second test set was omitted, since the investigated basis sets are not available for Zn. The required basis sets were taken from the Basis Set Exchange (BSE) library.^54–56^ The atomic-orbital (AO) basis sets utilized in this work were aug-cc-pVTZ-J,^57,58^ the pcJ-n series,^59^ and the pcH-n series,^2^ with n denoting the zeta quality. No relativistic effects were taken into account.

The resolution-of-the-identity (RI) was employed in both RPA and σ-functional calculations. Here, the correlation consistent basis-set series cc-pVXZ-RI (X ∈ {D,^60^ T,^60^ Q,^60^ 5 ^61^}) was employed for hydrogen together with cc-pwcVXZ-RI (X ∈ {D, T, Q, 5})^61^ for the remaining elements, except for Be, where cc-pwcV5Z-RI was always employed for availability reasons.

We further employed the RI for the calculation of the Coulomb contribution during the SCF (RI-J).^62^ Here, we used the cc-pVQZ-JKFIT^63^ auxiliary basis set. Since this basis set does not include Be, RI-J was not employed for Be-containing systems.

Analytical HFCCs at the RPA and σ-functional level of theory were implemented within our quantum chemistry package FermiONs ++.^64–66^ As starting points for the RPA calculations, several density functionals as provided by the Libxc library (version 5.1.1)^67^ have been tested. The investigated functionals include the generalized gradient approximation (GGA) of Perdew, Burke, and Ernzerhof (PBE),^68^ TPSS (*meta*-GGA),^69^ the hybrid *meta*-GGAs TPSSh and TPSS0 incorporating 10% and 25% HF exchange,^70^ respectively, the global hybrids PBE0 with 25% HF exchange^71,72^ and B3LYP with 20% HF exchange,^73^ as well as the range-separated hybrid functional ωB97X-V.^74^

For the σ-functionals, the W1 parametrization,^18^ which employs a larger number of reference sets and abscissa points than the original P parametrization,^17^ is available for PBE, TPSS, PBE0, and B3LYP. The S1 parametrization is based on systematically reworked reference sets and abscissa points, whereas the S2 parametrization is identical to S1 but additionally incorporates total energies with a small weight.^19^ Both S1 and S2 are implemented only for PBE and PBE0. All parametrization variants considered here were tested in this work.

The frozen core approximation was not utilized. All DFA calculations were carried out using a g7 numerical integration grid for both the DFA exchange–correlation parts and the seminumerical evaluation of exchange parts as reported in ref 75 to ensure an adequate description of the density. The SCF convergence criteria were 10^–8^ a.u. for the change in energy, 10^–7^ for the RMS of the FP-commutator, and 10^–7^ for the RMS of the difference density. Convergence was achieved only when all three criteria were satisfied simultaneously.

For the RPA-related contributions, we employed the linear-scaling RPA algorithm ω-CDGD-RI-RPA.^32^ Imaginary-time and -frequency integrations were performed using minimax grids with 20 grid points. Owing to the large AO basis sets used in this work, unusually large orbital energies were encountered in a number of cases, which occasionally led to numerical instabilities in the generation of the minimax grids. These issues were more pronounced for the (m)­GGA-based calculations, where small HOMO–LUMO gaps were also observed. In such cases, it was necessary to adjust the number of grid points and, where required, the singular value decomposition threshold used in the solution of the linear systems underlying the grid generation.

The coupled cluster calculations were performed using the ORCA program package (version 6.0.0).^76,77^ The CCSD­(T)-level hyperfine coupling constants were computed using an unrestricted Hartree–Fock (UHF) reference and relaxed densities. Again, the frozen core approximation was not employed. The SCF was converged employing the keyword *extreme* for the thresholds.

In addition, HFCCs were calculated with the domain-based local pair-natural orbital coupled cluster singles doubles (DLPNO–CCSD)^9^ approach. The auxiliary basis set was generated via the AutoAux technique. The keyword *DLPNO-HFC2* was used accompanied by the keyword *extreme* for the SCF to enforce tight truncation thresholds. All remaining settings were identical to those used for the CCSD­(T) calculations.

## 4 Results and Discussion

### 4.1 Small Main-Group Compounds

#### 4.1.1 Reference Values

First, we consider the test set of isotropic HFCCs for small main-group compounds published by Bartlett and co-workers.^5^ For these small systems (left column in Table 2) highly accurate CCSD­(T) results are obtainable, even with large basis sets. In the original work,^5^ the specialized aug-cc-pVTZ-J basis set was employed for the CCSD­(T) reference values. Aiming for properly converged results, we here test the pcJ^59^ and pcH^2^ basis-set series—optimized for J-couplings and HFCCs, respectively—introduced by the Jensen group. To ensure a fair comparison, we further recalculated the CCSD­(T) results using the aug-cc-pVTZ-J^57,58^ basis set with otherwise identical settings as for the calculations with the pcJ and pcH basis sets. We note that no Be basis functions are available for the pcH and aug-cc-pVTZ-J basis sets; therefore, the three Be-containing radicals were omitted in these cases. Furthermore, due to disk space limitations, isotropic HFCCs for F_2_CH, H_2_CCN, and H_2_CCO^+^ with the pcJ-4 basis set and for Cl_2_^–^, F_2_CH, CH_2_CH, H_2_CCN, and H_2_CCO^+^ with the pcH-4 basis set could not be obtained. The results are presented in Table 2.

**Table 2:** Isotropic Hyperfine Coupling Constants (MHz) for Small Main-Group Radicals Evaluated with CCSD­(T) Using Different Basis Sets

radical	nuclei	pcJ-1	pcJ-2	pcJ-3	pcJ-4	pcH-1	pcH-2	pcH-3	pcH-4	aug-cc-pVTZ-J
BO	^11^B	1007.5	1012.6	1026.4	1029.4	1017.4	1017.8	1027.5	1029.8	1017.5
	^17^O	–12.4	–13.7	–14.3	–14.5	–12.5	–13.7	–14.3	–14.5	–13.7
BeF	^9^Be	–293.0	–294.4	–285.6	–286.0					
	^19^F	279.1	228.4	218.8	218.5					
BeH	^9^Be	–187.2	–196.7	–192.8	–193.3					
	^1^H	254.1	211.2	202.1	199.4					
CH	^1^H	–62.0	–58.4	–58.1	–57.3	–62.0	–58.3	–58.6	–57.4	–59.2
	^13^C	23.6	32.1	41.2	41.9	23.4	32.9	41.2	42.0	39.6
CO^+^	^13^C	1519.1	1515.7	1532.9	1536.1	1534.4	1523.0	1534.1	1537.3	1521.5
	^17^O	18.9	19.7	19.5	19.3	19.5	19.8	19.5	19.4	20.2
Cl_2_^–^	^35^Cl	126.9	133.3	126.9	125.9	131.5	130.6	126.4		126.3
OH	^17^O	–50.8	–47.6	–51.6	–51.6	–50.9	–48.3	–51.6	–51.7	–50.0
	^1^H	–79.4	–74.0	–73.1	–72.5	–79.4	–74.0	–73.7	–72.6	–74.6
SH	^1^H	–58.5	–54.4	–52.6	–51.8	–58.4	–54.3	–53.1	–51.9	–54.1
	^33^S	27.6	37.1	35.9	36.5	34.3	34.8	35.3	36.2	36.2
BH_2_	^11^B	351.8	346.1	348.1	348.3	356.2	348.3	348.4	348.5	346.5
	^1^H	31.0	35.1	35.6	35.6	31.2	35.1	36.0	35.7	36.1
BeOH	^9^Be	–257.0	–266.2	–258.7	–259.1					
	^17^O	–51.8	–46.8	–46.2	–46.2					
	^1^H	2.2	2.4	2.5	2.5					
CH_2_	^13^C	245.2	235.2	236.9	236.7	248.3	236.8	237.1	236.9	235.8
	^1^H	–26.4	–21.5	–20.5	–20.2	–26.2	–21.4	–20.7	–20.2	–20.9
CH_2_^–^	^13^C	36.3	44.1	57.8	61.1	35.9	44.7	57.8	61.2	60.4
	^1^H	–53.4	–47.3	–44.7	–43.4	–53.4	–47.0	–45.1	–43.5	–44.2
C_2_H	^13^C	222.1	229.8	230.4	230.5	225.2	231.0	230.5	230.7	232.3
	^13^C	1006.1	1011.5	1023.1	1024.3	1017.5	1016.5	1023.8	1025.2	1017.4
	^1^H	52.5	49.5	48.7	48.4	53.0	49.5	49.1	48.5	49.8
HCO	^1^H	388.9	380.4	380.7	380.6	394.2	382.7	381.0	381.0	378.7
	^1^H	358.9	360.3	360.4	359.8	360.0	360.4	363.7	360.4	361.9
	^17^O	–47.1	–44.3	–44.7	–44.6	–47.5	–44.7	–44.7	–44.6	–44.5
HCS	^13^C	295.4	278.7	278.5	278.5	299.4	280.3	278.7	278.7	276.7
	^1^H	125.3	129.6	129.7	129.3	125.9	129.7	131.0	129.6	130.2
	^33^S	17.8	24.0	22.4	22.4	20.3	23.1	22.2	22.3	22.3
HOO	^1^H	–25.0	–24.0	–24.1	–24.0	–25.0	–24.0	–24.3	–24.0	–24.5
	^17^O1	–61.2	–57.0	–59.8	–59.7	–61.4	–57.8	–59.8	–59.7	–58.5
	^17^O2	–37.0	–34.2	–34.8	–34.8	–37.3	–34.6	–34.8	–34.8	–34.4
H_2_O^+^	^17^O	–92.5	–79.2	–80.3	–80.0	–93.5	–80.0	–80.3	–80.1	–79.3
	^1^H	–83.8	–77.2	–76.1	–75.6	–83.8	–77.1	–76.8	–75.7	–77.8
NH_2_	^1^H	–74.1	–67.9	–66.8	–66.2	–74.0	–67.8	–67.4	–66.3	–68.1
	^14^N	27.2	26.3	27.8	27.9	27.4	26.6	27.9	27.9	27.3
CH_3_	^13^C	82.7	72.7	74.2	74.1	83.7	73.7	74.2	74.2	73.2
	^1^H	–78.3	–71.7	–70.2	–69.7	–78.3	–71.6	–70.8	–69.8	–71.1
F_2_CH	^13^C	423.0	417.1	418.9		429.5	419.8	419.1		417.5
	^19^F	261.2	242.8	241.2		264.7	244.7	241.3		242.0
	^1^H	55.4	60.1	60.9		56.1	60.2	61.4		61.3
H_2_CN	^13^C	–80.6	–75.8	–75.5	–75.1	–81.6	–76.2	–75.5	–75.2	–76.3
	^1^H	213.4	213.8	215.7	215.9	213.5	213.8	217.6	216.2	215.5
	^14^N	24.5	23.6	25.0	25.0	24.6	23.9	25.0	25.0	24.4
NH_3_^+^	^14^N	50.5	45.3	45.7	45.5	51.3	45.7	45.7	45.6	45.5
	^1^H	–88.7	–82.5	–81.5	–81.0	–88.7	–82.3	–82.2	–81.1	–82.8
PH_3_^+^	^31^P	1200.2	1223.4	1186.0	1183.0	1212.8	1202.2	1183.4	1181.4	1193.8
	^1^H	–4.9	1.3	2.8	2.9	–5.2	1.3	2.7	2.9	2.1
CH_2_CH	^13^C	–16.5	–12.8	–12.8	–12.7	–16.6	–12.9	–12.8		–12.8
	^13^C	320.6	310.2	311.7	311.2	324.6	312.2	311.9		310.1
	^1^H	161.7	161.5	162.8	162.9	161.9	161.5	164.2		163.2
	^1^H	95.2	95.3	96.6	96.9	95.0	95.2	97.4		96.2
	^1^H	33.9	41.6	43.3	43.6	34.1	41.6	43.7		42.7
H_2_CCN	^13^CCH2	73.1	65.7	66.7		74.2	66.5	66.7		65.6
	^13^CCN	–65.8	–60.5	–60.0		–66.5	–60.9	–60.0		–60.7
	^1^H	–67.2	–61.7	–60.5		–67.2	–61.6	–61.1		–61.3
	^14^N	7.3	7.0	7.5		7.4	7.1	7.5		7.3
H_2_CCO^+^	^13^CCH2	69.6	62.2	63.2		70.9	63.0	63.2		62.3
	^13^CCO	–61.7	–58.3	–57.4		–62.2	–58.6	–57.4		–58.8
	^1^H	–67.3	–62.1	–60.9		–67.3	–62.0	–61.5		–61.8
	^17^O	–17.1	–14.8	–15.1		–17.2	–15.0	–15.1		–14.9

Inspecting Table 2, we first note that both the pcJ and pcH series generally exhibit systematic convergence with increasing cardinal number. However, there are notable exceptions—such as CO^+^, Cl_2_^–^, BeOH, and PH_3_^+^—where the HFCCs display nonmonotonic behavior. A closer inspection of the individual components revealed that these fluctuations originate from the underlying UHF reference wave function, whereas the electron correlation contributions themselves converge smoothly across the basis set series.

Furthermore, the convergence behavior of both series is overall very similar and the HFCCs obtained with the largest of both basis set families are in very close agreement. These findings not only show that both basis set families work very well with CC and probably also other wave function-based methods—although originally developed for density functionals^2^—but also suggest that the pcJ-4 and pcH-4 basis sets give results close to the complete basis set limit. Therefore, we are confident that we can continue our study with the pcJ basis set family. While choosing the pcJ basis sets over the pcH ones might seem counterintuitive, the pcJ basis set family is very attractive for practical applications because (1) they are available for more elements in the periodic system and (2) they are more contracted and hence more cost efficient than their pcH counterparts. Before continuing with basis-set extrapolations, we note that the CCSD­(T) results with the aug-cc-pVTZ-J basis set are not fully converged with respect to the basis-set size, which becomes most apparent for the large HFCCs.

To ensure a reliable discussion of method errors in the following sections, we try to rule out potential basis-set truncation errors as accurately as possible. We therefore test three different complete-basis-set (CBS) extrapolation schemes for the CCSD­(T) HFCCs obtained with the pcJ basis set series:

In scheme 1, we employ a standard two-point CBS extrapolation which is based on the universal cusp condition and typically utilized for correlation energies given by^78^

23 $$A_{\infty }=\frac{A_{X}X^{3}-A_{Y}Y^{3}}{X^{3}-Y^{3}}$$

Here, *A*_∞_ is the isotropic HFCC extrapolated to the CBS limit and *X* as well as *Y* denote the cardinal numbers of the basis sets used to calculate the HFCCs *A*_*X*_ and *A*_*Y*_, respectively. In scheme 2, we use a two-point extrapolation which was employed by Bartlett and co-workers^79^ for extrapolating HFCCs to the CBS limit

24 $$A_{X}=A_{\infty }+Be^{-\left(X-1\right)}$$

Since scheme 2 was proposed as a two-point approximation of the extrapolation formula presented by Dunning and co-workers,^80^ we will present results obtained with this third extrapolation scheme as well

25 $$A_{X}=A_{\infty }+Be^{-\left(X-1\right)}+Ce^{-{\left(X-1\right)}^{2}}$$

The results presented in Table 3 show that, overall, the three different extrapolation schemes utilizing the pcJ-4 data give very similar results. In fact, the pcJ-[3,4] variant of scheme 2 and pcJ-[2,3,4] (scheme 3) give, up to the second decimal place, identical results for all but three HFCCs (for which the deviation is only 0.1 MHz), underlining the reliability of the two-point approximation proposed by Bartlett and co-workers.^79^ Furthermore, also the pcJ-[2,3] and pcJ-[3,4] variants of schemes 1 and 2 are in good agreement. Since the pcJ-[2,3] variants of schemes 1 and 2 agree very well for the systems for which no pcJ-4 data is available, we are confident that the pcJ-[2,3] results serve as accurate references in these cases. Thus, scheme 2 pcJ-[3,4] results will be used as reference values wherever possible in the following, supplemented by the pcJ-[2,3] results for F_2_CH, H_2_CCN, and H_2_CCO^+^.

**Table 3:** Experimental and CBS-Extrapolated CCSD­(T) Isotropic Hyperfine Coupling Constants (MHz) for Small Main-Group Radicals Obtained Using Various Extrapolation Schemes^a^

radical	nuclei	exp	scheme 1			scheme 2			scheme 3
			pcJ-[2,3]	pcJ-[3,4]	error	pcJ-[2,3]	pcJ-[3,4]	error	pcJ-[2,3,4]
BO	^11^B	1024.9	1036.6	1032.4	0.4	1034.5	1031.1	0.3	1031.0
	^17^O	14.0	–14.7	–14.8	0.2	–14.7	–14.7	0.0	–14.7
BeF	^9^Be	294.0	–279.1	–286.4	2.6	–280.4	–286.2	2.0	–286.2
	^19^F	229.0	211.8	218.1	3.0	213.2	218.3	2.4	218.3
BeH	^9^Be	199.3	–190.1	–193.8	2.0	–190.6	–193.6	1.6	–193.6
	^1^H	193.9	195.4	196.6	0.6	196.8	197.9	0.5	197.9
CH	^1^H	57.7	–57.8	–56.5	2.2	–57.9	–56.9	1.7	–56.9
	^13^C	47.1	47.9	42.7	10.9	46.5	42.3	9.0	42.3
CO^+^	^13^C	1573.0	1545.5	1539.5	0.4	1543.0	1538.0	0.3	1538.0
	^17^O	18.5	19.4	19.2	1.1	19.4	19.2	0.8	19.2
Cl_2_^–^	^35^Cl	109.0	122.2	124.8	2.1	123.2	125.3	1.7	125.3
OH	^17^O	51.3	–54.5	–51.7	5.1	–53.9	–51.7	4.1	–51.7
	^1^H	71.5	–72.4	–71.9	0.7	–72.6	–72.2	0.5	–72.2
SH	^1^H	65.0	–51.3	–51.0	0.5	–51.5	–51.4	0.3	–51.4
	^33^S		35.0	37.2	6.2	35.2	36.9	4.8	36.9
BH_2_	^11^B	357.9	349.6	348.5	0.3	349.3	348.4	0.2	348.4
	^1^H	38.0	36.0	35.5	1.4	35.9	35.5	1.1	35.5
BeOH	^9^Be	264.0	–253.2	–259.5	2.5	–254.3	–259.3	2.0	–259.3
	^17^O		–45.9	–46.1	0.5	–45.9	–46.1	0.4	–46.1
	^1^H	<5.0	2.5	2.4	4.8	2.5	2.4	3.8	2.4
CH_2_	^13^C		238.2	236.5	0.7	237.9	236.6	0.6	236.6
	^1^H	20.2	–19.8	–19.9	0.5	–20.0	–20.1	0.5	–20.1
CH_2_^–^	^13^C	58.9	67.8	64.6	4.7	65.8	63.1	4.1	63.1
	^1^H	44.8	–42.8	–42.2	1.4	–43.1	–42.7	1.0	–42.7
C_2_H	^13^C	213.0	230.9	230.6	0.1	230.8	230.5	0.1	230.5
	^13^C	1014.5	1031.5	1025.6	0.6	1029.8	1025.0	0.5	1025.0
	^1^H	50.4	48.2	48.1	0.0	48.3	48.3	0.0	48.3
HCO	^1^H	377.5	381.0	380.5	0.1	380.9	380.6	0.1	380.6
	^1^H	354.0	360.4	359.1	0.4	360.4	359.4	0.3	359.4
	^17^O	42.3	–44.9	–44.5	1.0	–44.9	–44.5	0.8	–44.5
HCS	^13^C		278.3	278.5	0.1	278.3	278.5	0.1	278.5
	^1^H	127.5	129.7	128.9	0.6	129.7	129.1	0.5	129.1
	^33^S		21.2	22.4	5.5	21.4	22.4	4.3	22.4
HOO	^1^H	27.4	–24.1	–23.8	1.1	–24.1	–23.9	0.9	–23.9
	^17^O1		–61.8	–59.6	3.5	–61.4	–59.6	2.8	–59.6
	^17^O2		–35.1	–34.8	1.0	–35.1	–34.8	0.8	–34.8
H_2_O^+^	^17^O	83.2	–81.0	–79.7	1.6	–80.9	–79.8	1.3	–79.8
	^1^H	73.1	–75.3	–75.0	0.5	–75.5	–75.2	0.4	–75.2
NH_2_	^1^H	67.2	–65.9	–65.6	0.5	–66.1	–65.9	0.3	–65.9
	^14^N	27.9	29.0	27.9	3.7	28.8	27.9	2.9	27.9
CH_3_	^13^C	75.7	75.3	74.0	1.7	75.1	74.0	1.4	74.0
	^1^H	70.1	–69.1	–69.1	0.1	–69.3	–69.4	0.1	–69.4
F_2_CH	^13^C	417.0	420.1			419.9			
	^19^F	236.0	240.1			240.3			
	^1^H	62.2	61.4			61.3			
H_2_CN	^13^C	81.0	–75.2	–74.8	0.6	–75.3	–74.9	0.4	–74.9
	^1^H	233.2	217.0	216.2	0.4	216.7	216.1	0.3	216.1
	^14^N	25.8	26.0	25.0	3.9	25.8	25.0	3.2	25.0
NH_3_^+^	^14^N	54.9	46.0	45.4	1.3	45.9	45.5	1.0	45.4
	^1^H	76.8	–80.8	–80.4	0.4	–80.9	–80.7	0.3	–80.7
PH_3_^+^	^31^P	1176.0	1158.7	1179.8	1.8	1164.3	1181.2	1.5	1181.3
	^1^H	<5.9	3.8	3.1	20.1	3.6	3.0	17.2	3.0
CH_2_CH	^13^C	24.0	–12.9	–12.6	1.8	–12.8	–12.7	1.4	–12.7
	^13^C	301.5	312.7	310.8	0.6	312.5	311.0	0.5	311.0
	^1^H	192.0	163.7	163.1	0.3	163.5	163.0	0.3	163.0
	^1^H	95.8	97.5	97.1	0.4	97.3	97.0	0.3	97.0
	^1^H	37.3	44.6	43.9	1.6	44.4	43.8	1.3	43.8
H_2_CCN	^13^CCH2		67.4			67.2			
	^13^CCN		–59.6			–59.7			
	^1^H	58.8	–59.6			–59.8			
	^14^N	9.8	7.8			7.8			
H_2_CCO^+^	^13^CCH2		63.9			63.8			
	^13^CCO		–56.7			–56.9			
	^1^H	58.0	–60.1			–60.2			
	^17^O		–15.3			–15.3			
MAE [MHz]			6.59	6.33		6.36	6.35		6.35
MAPE [%]			6.08	6.08		5.92	5.97		5.97

a Relative errors of the pcJ-[2,3] extrapolations with respect to the pcJ-[3,4] extrapolations are given in %. Mean absolute errors (MAE) and mean absolute percentage errors (MAPE) of the CBS-extrapolated CCSD­(T) HFCCs relative to experiment are also reported.

Before discussing the performance of RPA and σ-functionals in the next section, we note that even the CBS extrapolated CCSD­(T) results show a mean absolute error (MAE) of around 6 MHz with respect to the experimental results. The source of these deviations is probably a combination of missing dynamic and solvent effects as well as remaining method errors. The importance of these effects for obtaining accurate HFCCs has already been discussed in the literature;^7,12,81^ however, more detailed investigations along these lines are beyond the scope of the present work.

#### 4.1.2 Performance of RPA and σ-functionals

In this section, we investigate the accuracy of isotropic HFCCs for the 26 small main-group compounds shown in Tables 2 and 3 evaluated with RPA and the related σ-functionals by comparing with the CCSD­(T) reference values established in the previous section. Since, in this work, we utilize the RPA and the σ-functionals as typical post-KS methods, we further investigate their dependence on the reference DFA calculations. For comparison, we also present results obtained with B3LYP, as the functional was shown to perform well for the computation of HFCCs and is further commonly employed for that purpose in the quantum chemistry community. As mentioned earlier, the DLPNO–CCSD method is, at the moment, probably the most accurate method for HFCC computations of systems beyond the few-atoms scale. We therefore also include DLPNO–CCSD results in our discussion to have a direct comparison.

Figure 1 shows the MAEs of the various methods utilizing the pcJ basis-set series and the same basis-set extrapolation (scheme 2) as utilized for the CCSD­(T) method in the previous section. Note that we always compare with the CBS extrapolated CCSD­(T) results and hence basis-set errors will contribute to the shown MAEs.

**Figure 1:**
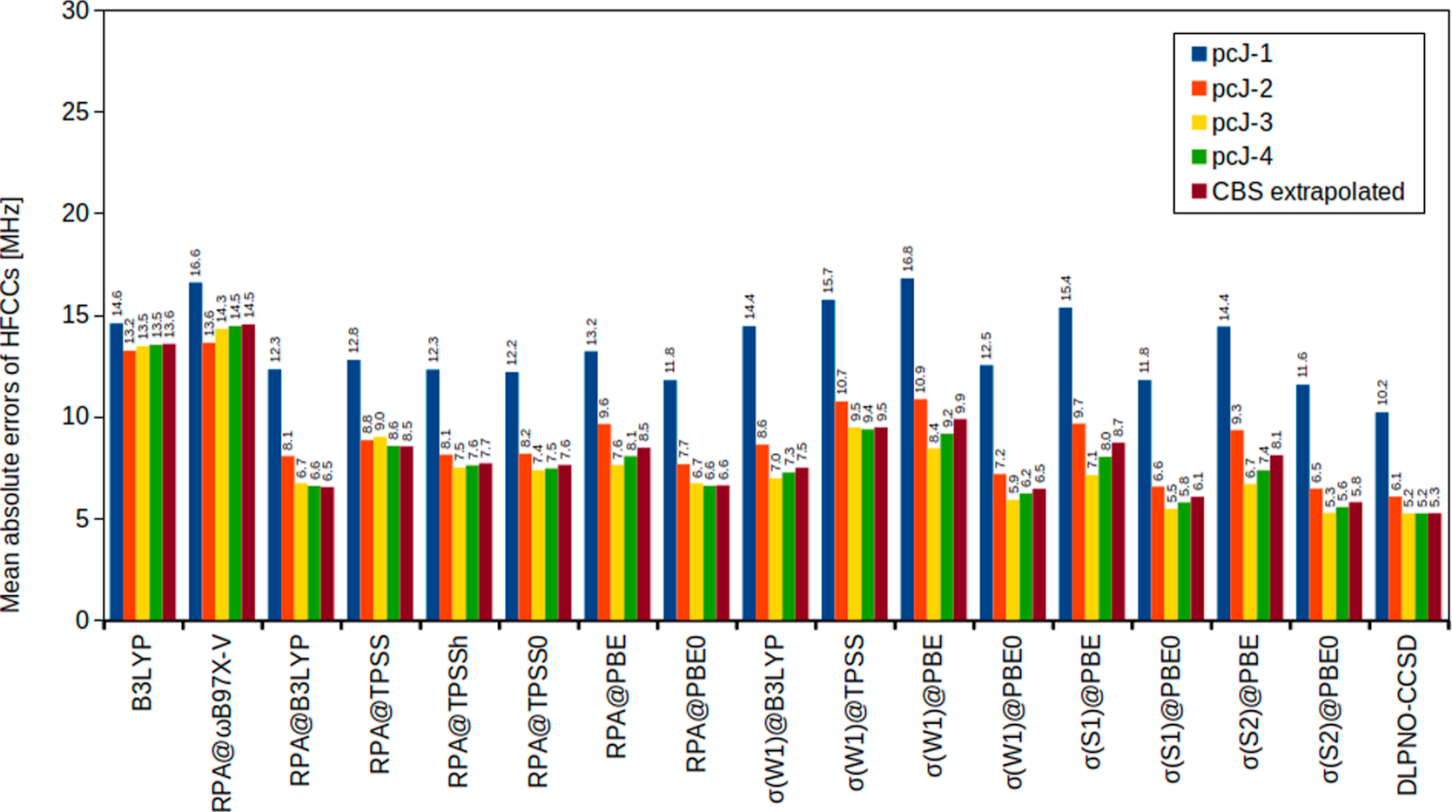
Mean absolute errors (MHz) of isotropic HFCCs for small main-group compounds using the pcJ basis sets compared to CBS extrapolated CCSD­(T) values.

Starting with B3LYP, we observe an excellent convergence of the MAEs with respect to the basis-set size. While this is expected since the B3LYP functional was employed in the development of this basis set family,^2^ B3LYP performs significantly worse than almost all other methods tested in this work. The only exception is RPA based on ωB97X-V reference orbitals and orbital energies, denoted as RPA@ωB97X-V, which performs slightly worse than B3LYP. This is a notable observation, given that ωB97X-V is generally considered to be among the best hybrid functionals available;^82,83^ i.e., it does not seem to be suitable in combination with the RPA—at least not for the computation of HFCCs.

Apart from RPA@ωB97X-V, RPA performs very well and shows only a weak dependence on the reference DFA, as was also reported by Franzke and co-workers.^39^ It further shows that the RPA results converge excellently with respect to the basis-set size, with the pcJ-3 basis set typically being the sweet-spot. Therefore, the pcJ basis-set family can clearly be recommended for the computation of RPA HFCCs. The best performing RPA variant is RPA@B3LYP and hence our recommendation in combination with the pcJ-3 basis set.

Turning toward the σ-functionals, they overall show similar performance as the RPA variants. Regarding the parametrization, the latest S2 parametrization^19^ generally shows the highest accuracy. In terms of the reference DFA, PBE0 clearly stands out, leading us to our recommendation of the σ­(S2)@PBE0 functional. The basis-set dependence is slightly more pronounced than for all other tested methods. Moreover, the results seem to deteriorate with basis sets larger than the pcJ-3 variant, which is again our recommendation. The reason for that behavior might be that σ-functionals are optimized together with finite basis sets and hence the parametrization might account to some small extent for basis-set incompleteness errors.

DLPNO–CCSD yields the lowest MAE among all tested methods and further shows excellent convergence with respect to the pcJ basis-set size, with pcJ-3 again being the sweet-spot. However, the DLPNO–CCSD method is by far the most computationally demanding method in our test suite. As the MAE is only slightly lower than that of σ­(S2)@PBE0 (0.2 MHz), σ­(S2)@PBE0 is an excellent alternative to DLPNO–CCSD.

In addition to the MAEs, we present the respective mean absolute percentage errors (MAPEs) in Figure 2. Using this metric to gauge the quality of our results is insightful because the MAEs tend to be dominated by large HFCCs while MAPEs tend to be dominated by small HFCCs, giving a different perspective on the performance of the methods.

**Figure 2:**
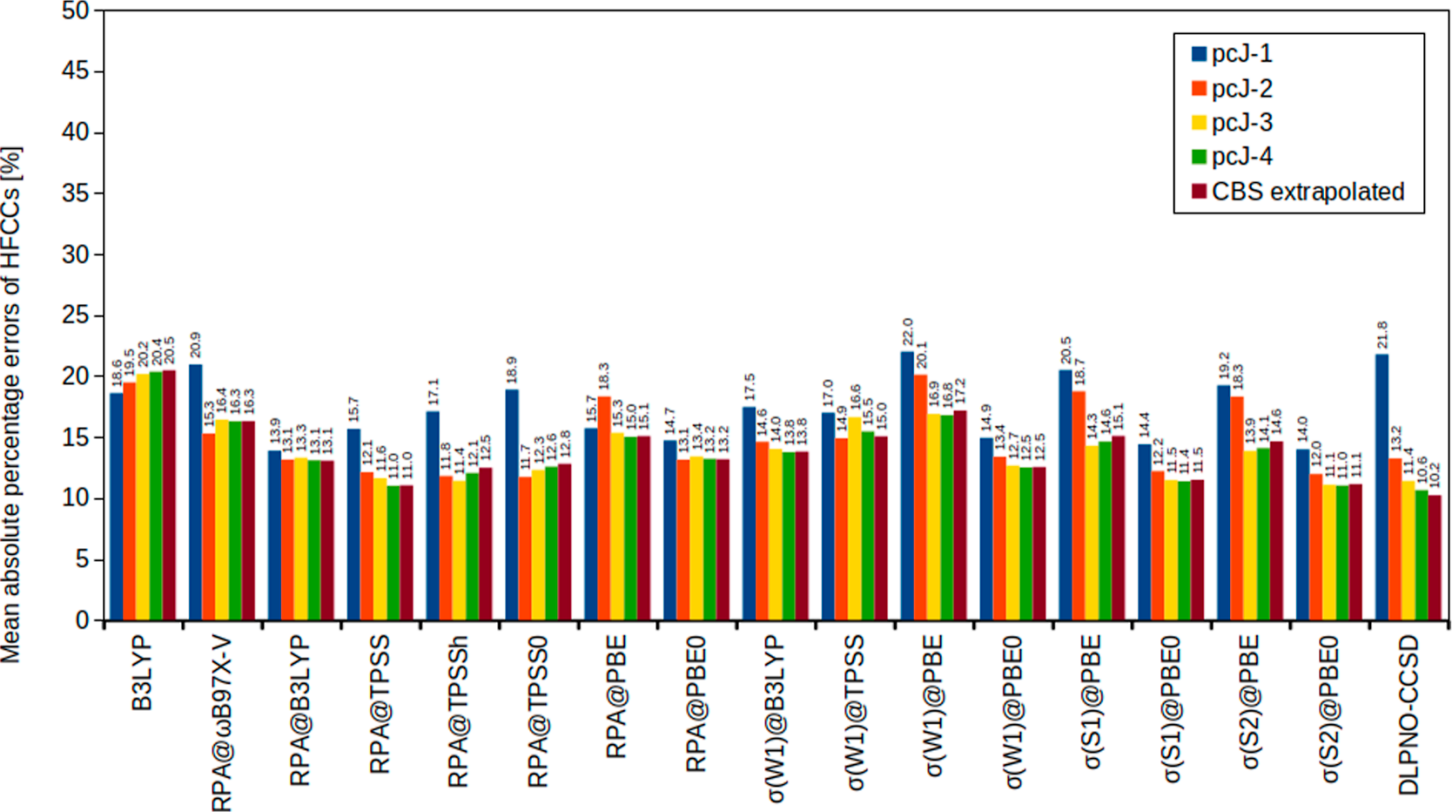
Mean absolute percentage errors (%) of isotropic HFCCs for small main-group compounds using the pcJ basis sets compared to CBS extrapolated CCSD­(T) values.

Still, our conclusions from analyzing the different MAEs are mostly unaltered. The main difference is that, from the MAPE perspective, RPA@TPSSh should be preferred over RPA@B3LYP while the pcJ-3 basis remains our recommendation. Although RPA@TPSSh is now on the same level of accuracy as the best performing σ-functional—again σ­(S2)@PBE0—we would still recommend σ-functionals over a standard RPA treatment as, on the bigger picture, σ­(S2)@PBE0 gives more consistent results and further, the additional computational cost of evaluating a σ-functional is marginal compared to a standard RPA calculation. Another noteworthy point concerns the basis-set convergence: The σ-functional results shown in Figure 2 converge significantly better. Also, DLPNO–CCSD does benefit from a CBS extrapolation; however, the moderate increase in accuracy might not justify the significant increase in computational cost.

#### 4.1.3 Influence of the Auxiliary Basis Set

Both RPA and the related σ-functionals rely on a resolution-of-the-identity (RI) approach for computational efficiency reasons. In this section, we hence investigate the dependence of the results obtained with these approaches on the size of the chosen auxiliary basis set. As representatives, we opted for the two best-performers in terms of MAEs from the CBS extrapolated CCSD­(T) reference values, RPA@B3LYP and σ­(S2)@PBE0, in combination with the pcJ-4 basis set. Note, however, that the three Be-containing species (BeH, BeF, and BeOH) are excluded from this analysis because only the cc-pwcV5Z-RI auxiliary basis includes basis functions for beryllium. The results are shown in Figure 3.

**Figure 3:**
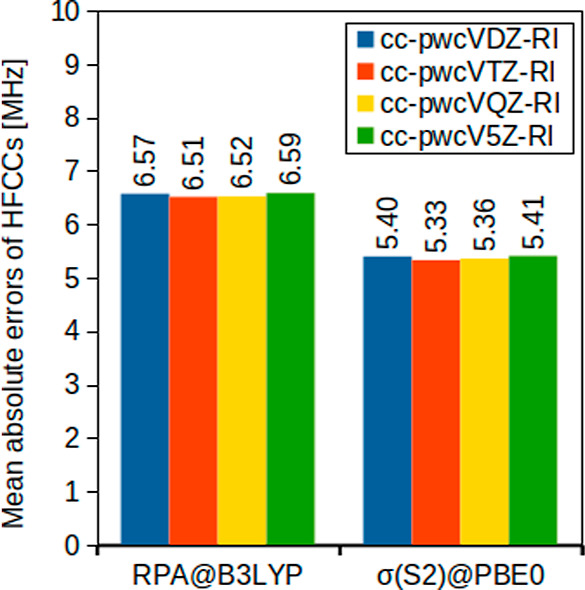
Mean absolute errors (MHz) of isotropic HFCCs for small main-group compounds evaluated with the pcJ-4 and different auxiliary basis sets compared to CBS-extrapolated CCSD­(T) values.

It is evident from the similar MAEs that the influence of the auxiliary basis is very small. Since the time-determining step in the calculation of RPA and σ-functional HFCCs scales as *N*_aux_^2^ *N*_basis_ *N*_occ_, the computational cost can be reduced significantly by utilizing a smaller auxiliary basis. In light of this, cc-pwcVTZ-RI offers a suitable balance between accuracy and efficiency and is hence our recommendation for use together with the pcJ basis sets.

### 4.2 Large Main-Group Compounds

In this section, we compare isotropic HFCCs of large main-group radicals^5^ computed with the B3LYP functional, RPA@B3LYP, RPA@PBE0, σ­(S2)@PBE0, and DLPNO–CCSD against CCSD results taken from ref 5 and experimental results, where available. We note that these comparisons are far from ideal since (1) we only have CCSD and no CCSD­(T) reference values available, (2) these CCSD values were computed with the aug-cc-pVTZ-J basis set and are hence likely not fully converged (see Table 2), and (3) experimental results are always difficult to compare against due to the lack of dynamic and solvent effects in the calculations (see Table 3). The results of this section are summarized in Table 4.

**Table 4:** pcJ-[2,3] Extrapolated Isotropic Hyperfine Coupling Constants (MHz) for Large Main-Group Radicals Computed with Different Methods

radical	nuclei	exp	CCSD^a^	B3LYP	RPA@B3LYP	RPA@PBE0	σ(S2)@PBE0	DLPNO–CCSD
aniline+	^13^C1, ^13^C5		–25.2	–19.0	–22.6	–23.9	–20.9	–27.6
	^13^C2, ^13^C4		12.5	12.1	12.8	13.4	11.8	15.7
	^13^C3		–19.4	–13.1	–15.8	–17.0	–13.3	–19.2
	^13^C6		35.2	32.3	35.6	36.4	34.6	36.5
	^1^ H7, ^1^ H10	4.3	4.0	3.9	3.2	3.7	1.9	5.2
	^1^H8,^1^H9	16.3	–15.8	–14.2	–14.1	–14.6	–12.7	–16.7
	^1^H11	26.8	–33.8	–28.4	–29.9	–31.1	–28.3	–34.4
	^1^H13, ^1^H14	26.8	–28.7	–26.5	–27.6	–28.1	–25.1	–27.2
	^14^N12	21.5	17.2	14.6	17.9	18.1	16.7	16.6
4-nitroaniline+	^13^C1, ^13^C5		–28	–20.3	–24.3	–25.0	–21.4	–29.3
	^13^C2, ^13^C4		16.4	14.4	15.4	15.9	13.8	19.3
	^13^C3		–24.2	–16.2	–19.4	–20.0	–15.5	–22.7
	^13^C6		48.8	40.0	46.7	47.3	43.8	49.0
	^1^H7, ^1^H10	5.8	6.5	5.7	4.8	4.9	2.5	7.3
	^1^H8, ^1^H9	18.0	–17.9	–15.3	–15.4	–15.5	–13.1	–18.4
	^1^H12, ^1^H13	28.7	–29.2	–26.6	–28.0	–28.1	–24.9	–27.2
	^14^N11	22.4	17.6	14.6	18.1	18.2	16.7	17.3
	^14^N14	5.8	–7.6	–5.3	–6.5	–6.7	–5.9	–7.9
	^17^O15, ^17^O16		–2.1	–2.0	–2.6	–2.6	–2.7	–2.0
benzyl	^13^C1, ^13^C2		22.9	20.4	22.8	23.8	21.2	23.4
	^13^C3, ^13^C5		–22.5	–18.5	–21.3	–22.5	–19.5	–23.9
	^13^C4		23.8	21.7	24.0	24.7	22.3	23.0
	^13^C11	40.5	–50.4	–38.0	–46.3	–48.9	–44.5	–48.9
	^13^C12	68.5	63.6	60.5	67.3	68.1	65.6	62.7
	^1^H6, ^1^H7	14.4	–15.9	–14.8	–14.5	–15.4	–13.3	–16.1
	^1^H8, ^1^H10	5.0	6.1	6.1	5.6	6.3	4.4	6.8
	^1^H9	17.3	–19.5	–17.5	–17.8	–18.7	–16.6	–19.2
	^1^H13, ^1^H14	45.7	–55.1	–48.1	–51.5	–54.2	–50.3	–54.8
phenylaminyl	^13^C1		–27.7	–21.2	–25.6	–26.1	–22.8	–28.1
	^13^C2		25.6	21.7	24.8	25.1	22.3	26.5
	^13^C3		–50.8	–37.9	–45.8	–46.8	–41.6	–49.1
	^13^C4		24.1	20.7	23.9	24.2	21.5	25.1
	^13^C5		–27.9	–21.5	–25.8	–26.3	–23.0	–28.3
	^13^C6		30.4	26.2	30.1	30.3	28.0	28.0
	^1^H7	5.6	7.9	7.0	6.9	6.9	4.7	7.9
	^1^H8	17.3	–19.5	–17.0	–17.6	–17.7	–15.4	–18.9
	^1^H9	17.3	–19.7	–17.3	–17.9	–18.0	–15.6	–19.0
	^1^H10	5.6	8	7.1	7.1	7.1	4.9	8.0
	^1^H11	23.0	–24.8	–21.1	–22.9	–23.1	–20.9	–23.6
	^1^H13	36.3	–45.6	–39.6	–41.2	–42.2	–38.4	–45.7
	^14^N12	22.3	24.5	21.1	25.6	25.8	24.7	24.5
1,3,2-benzodithiazolyl	13C1, 13C2		–7.1	–5.0	-5.9	-6.3	-4.8	–7.4
	^13^C3, ^13^C6		0.0	0.8	0.2	0.1	0.4	0.0
	^13^C4, ^13^C5		–0.5	–0.2	–0.3	–0.3	–0.3	0.0
	^1^H7, ^1^H10	–1.7	–1.3	–1.8	–1.4	–1.2	–1.7	–1.1
	^1^H8, ^1^H9	–1.7	–1.2	–1.5	–1.4	–1.4	–1.5	–1.1
	^33^S11, ^13^S12	10.9	9.9	5.6	10.3	10.6	8.0	5.3
	^14^N13	30.8	36.3	26.9	34.5	33.7	33.0	35.6
diethylaminyl	^13^C1, ^13^C9		–2.4	–2.0	–1.7	–1.8	–1.7	–2.1
	^13^C2, ^13^C8		–37.9	–29.7	–35.5	–35.8	–35.2	–38.0
	^1^H3, ^1^H12		–3.6	–3.1	–3.2	–3.3	–3.0	–3.6
	^1^H4, ^1^H5, ^1^H13, ^1^H14		–1.8	–1.5	–1.8	–1.8	–1.6	–1.9
	^1^H6, ^1^H7, ^1^H10, ^1^H11	103.4	98.4	112.1	97.7	98.4	102.3	100.2
	^14^N15	40.0	40.3	34.4	42.5	42.0	41.4	38.4
cyclo-hexyl	^13^C1		–1.8	–0.7	–1.9	–2.0	–1.6	–1.7
	^13^C2, ^13^C6		21.9	23.6	21.8	21.9	22.3	22.5
	^13^C3, ^13^C5		–33.1	–24.2	–31.2	–31.9	–30.7	–33.5
	^13^C4	115.7	119.9	114.7	125.7	125.9	123.8	117.8
	^1^H7, ^1^H13	14.9	15.5	17.3	15.5	15.8	15.9	15.9
	^1^H8, ^1^H15	2.0	–3.1	–2.5	–2.9	–2.9	–2.8	–3.0
	^1^H9, ^1^H16	2.0	–0.1	0.9	–0.1	–0.1	0.2	–0.1
	^1^H10		–0.1	0.1	–0.1	–0.1	0.0	0.0
	^1^H11		2.2	2.8	2.0	2.1	2.2	2.2
	^1^H12	59.7	–63.7	–54.4	–61.3	–63.0	–59.1	–63.8
	^1^H14, ^1^H17	110.4	117.5	130.3	116.8	117.3	119.5	118.7
1-adamantyl	^13^C1, ^13^C7, ^13^C21		–23.1	–16.9	–20.2	–21.1	–19.7	–22.4
	^13^C4, ^13^C13, ^13^C24		38.8	38.5	39.0	39.0	38.8	38.8
	^13^C6		223.4	211.1	224.5	226.2	222.5	222.0
	^13^C10, ^13^C15, ^13^C18		–4.7	–3.9	–4.4	–4.5	–4.3	–4.2
	^1^H2, ^1^H3, ^1^H8, ^1^H9, ^1^H22, ^1^H23	18.4	17.5	19.7	17.1	17.4	17.5	17.8
	^1^H5, ^1^H14, ^1^H25	13.1	10.9	13.1	11.3	11.2	11.6	11.0
	^1^H11, ^1^H17, ^1^H20	8.6	6.7	9.0	6.6	6.6	7.2	6.3
	^1^H12,^1^H16, ^1^H19	2.2	1.9	2.4	1.8	1.8	1.9	1.8
MAE to CCSD [MHz]				3.75	1.39	1.11	2.52	0.78
MAE to exp. [MHz]			2.83	2.68	2.21	2.45	2.34	2.92

a aug-cc-pVTZ-J.

Starting with the comparison against CCSD results, we observe that, as expected, DLPNO–CCSD gives the lowest MAE (0.8 MHz). We note that the source of this deviation is a combination of the DLPNO-approximation and the basis-set incompleteness in case of the CCSD reference values. More surprisingly, RPA@B3LYP gives a MAE (1.4 MHz) which is almost half the MAE of the best performer for small main-group radicals, σ­(S2)@PBE0 (2.5 MHz).

To get an idea whether the base-functional, PBE0, could be the reason for the decreased accuracy of σ­(S2)@PBE0 on this test set, we computed isotropic HFCCs with RPA@PBE0. As shown in Table 4, RPA@PBE0 outperforms RPA@B3LYP when CCSD is used as the reference (MAE: 1.1 MHz). Although error cancellations are possible, it can be assumed that the PBE0 reference is likely not the cause of the deteriorated performance of σ­(S2)@PBE0.

Turning to the comparison with experimental values, we first note that experimental HFCCs are available for every single *system*, however, not for every single *nucleus*. This means that, with the limitations mentioned above in mind, the comparison still gives an idea of the methods’ performance for the different systems at hand.

Interestingly, we observe that CCSD (MAE: 2.8 MHz) and DLPNO–CCSD (MAE: 2.9 MHz) show worse agreement with the experimental values than RPA@B3LYP (MAE: 2.2 MHz) and σ­(S2)@PBE0 (MAE: 2.3 MHz). Furthermore, the difference between the MAEs of RPA@B3LYP and σ­(S2)@PBE0 is, in this case, significantly smaller. We note that we also evaluated the MAEs of RPA@B3LYP and σ­(S2)@PBE0 HFCCs with respect to the CCSD reference values for the experimentally accessible nuclei and we found the same trend as for the complete test set: RPA@B3LYP performs significantly better than σ­(S2)@PBE0. This means that it is not the selection of nuclei but the reference values which cause this change in agreement. Again, error compensation could be the reason for these observations; however, it is also possible that the σ-functional gives higher accuracies as could be assumed from the comparison with CCSD reference values. Unfortunately, this question remains unanswered until better reference values are available.

To provide insight into the relative computational cost of the different approaches, we compared their execution times using 1-adamantyl—the largest system in the test set—as an illustrative example. All methods employed the pcJ-2 and pcJ-3 atomic-orbital basis sets, as well as the cc-pVQZ-JKFIT auxiliary basis set for RI-J. For the RPA, σ-functional, and DLPNO–CCSD calculations, the cc-pwcV5Z-RI auxiliary basis set was additionally used in the correlation treatment.

The calculations were performed on a single compute node equipped with two AMD EPYC 7302 CPUs (64 cores total) and 256 GB of memory. A key practical advantage of the σ-functional and RPA implementations is their modest memory footprint, which allowed us to fully exploit all 64 available cores. In contrast, the inherently higher memory-*per*-core requirements of the DLPNO–CCSD method restricted its parallelization to 8 processes on this specific hardware.

For convergence and truncation tolerances, we relied on ORCA’s built-in macro keywords (e.g., *ExtremeSCF* and *DLPNO-HFC2*) rather than manually fine-tuning individual screening parameters to maximize computational performance (refer to the Supporting Information for the input files). Consequently, the presented DLPNO–CCSD timings are intended to provide an order-of-magnitude baseline on standard hardware, rather than an optimized benchmark of the DLPNO–CCSD implementation itself.

The resulting wall-clock timings for the different methods and basis sets are summarized in Table 5. Identical computation times are observed for both the RPA methods and the σ-functional. Furthermore, their computational cost is on the same order of magnitude as the standard hybrid DFT calculations using the B3LYP functional. In contrast, the DLPNO–CCSD calculations result in a substantial increase in computation time.

**Table 5:** Comparison of Computational Wall-Clock Timings (in Hours) for HFCC Calculations of 1-Adamantyl Across Various Methods and Basis Sets, Including the Number of Processor Cores Utilized Based on Hardware Memory Constraints

	B3LYP	RPA@B3LYP	RPA@PBE0	σ(S2)@PBE0	DLPNO–CCSD
cores	64	64	64	64	8
					
pcJ-2	0.1	0.2	0.2	0.2	12.3
pcJ-3	0.3	0.6	0.6	0.6	58.1

## 5 Conclusion

The random-phase approximation (RPA) and the closely related σ-functionals have proven to be highly promising approaches for the computation of accurate hyperfine coupling constants at a moderate computational cost (on the order of minutes for the systems and basis sets considered in this work). For both RPA and σ-functionals the use of hybrid functionals to generate the input orbitals is recommended. In particular, B3LYP and PBE0 performed best in combination with RPA; for σ-functionals PBE0 showed to be the best choice. For the latter, the choice of parametrization does have a noticeable impact, with the most recent S2 parametrization giving the highest accuracy.

Concerning the atomic-orbital basis, the pcH and pcJ basis-set families developed by the Jensen group are clearly recommended for all electronic-structure methods employed in this work, including RPA, σ-functionals, and (DLPNO) coupled-cluster approaches. Both basis-set families yield very similar accuracies, however, due to its higher degree of contraction and correspondingly lower computational cost, the pcJ family appears preferable in practice. Our results further indicate that complete-basis-set extrapolations offer no meaningful benefit for RPA and σ-functionals, as any accuracy gains are marginal relative to the increased computational effort. The pcJ-3 basis therefore represents a well-balanced and robust choice. The dependence of the RPA and σ-functional results on the auxiliary basis was found to be weak, with cc-pwcVTZ-RI being sufficient.

Overall, RPA@B3LYP, RPA@PBE0, and σ­(S2)@PBE0 in combination with the pcJ-3 atomic-orbital basis and the cc-pwcVTZ-RI auxiliary basis are highly promising approaches for the computation of accurate hyperfine coupling constants. These methods represent strong and practical alternatives to the substantially more expensive (DLPNO−)­CCSD methods and we therefore expect that they will find broad application in future studies of hyperfine couplings.

## Supplementary Material



## A **H** ^σ^ and Its Derivative with Respect to σ

For simplicity, σ denotes an element of the diagonal eigenvalue matrix **σ**(iω) for a given imaginary frequency. **H**(σ) is defined as^18^

26
$$H\left(\sigma \right)=\{\begin{array}{ll} 0 & |\sigma \in I_{1}\\ c_{0,2}\left(\sigma -x_{2}\right)+\frac{1}{2}c_{1,2}{\left(\sigma -x_{2}\right)}^{2}+\frac{1}{3}c_{2,2}{\left(\sigma -x_{2}\right)}^{3}+\frac{1}{4}c_{3,2}{\left(\sigma -x_{2}\right)}^{4} & |\sigma \in I_{2}\\ \sum_{i=2}^{m-1}h_{i}+c_{0,m}\left(\sigma -x_{m}\right)+\frac{1}{2}c_{1,m}{\left(\sigma -x_{m}\right)}^{2}+\frac{1}{3}c_{2,m}{\left(\sigma -x_{m}\right)}^{3} & \\ +\frac{1}{4}c_{3,m}{\left(\sigma -x_{m}\right)}^{4} & |\sigma \in I_{m};m\in \left[3,M-2\right]\\ \sum_{i=2}^{M-2}h_{i} & |\sigma \in I_{M-1},I_{M} \end{array}$$

with

27
$$h_{i}=c_{0,i}\Delta x_{i}+\frac{1}{2}c_{1,i}\Delta x_{i}^{2}+\frac{1}{3}c_{2,i}\Delta x_{i}^{3}+\frac{1}{4}c_{3,i}\Delta x_{i}^{4}$$

28
$$\Delta x_{i}=x_{i+1}-x_{i}$$

Its derivative with respect to σ is given by

29
$$\frac{\partial H^{\sigma }\left(\sigma \right)}{\partial \sigma }=\{\begin{array}{ll} 0 & |\sigma \in I_{1}\\ c_{0,2}+c_{1,2}\left(\sigma -x_{2}\right)+c_{2,2}{\left(\sigma -x_{2}\right)}^{2}+c_{3,2}{\left(\sigma -x_{2}\right)}^{3} & |\sigma \in I_{2}\\ c_{0,m}+c_{1,m}\left(\sigma -x_{m}\right)+c_{2,m}{\left(\sigma -x_{m}\right)}^{2}+c_{3,m}{\left(\sigma -x_{m}\right)}^{3} & |\sigma \in I_{m};m\in \left[3,M-2\right]\\ 0 & |\sigma \in I_{M-1},I_{M} \end{array}$$

## Notes

The Supporting Information contains all hyperfine coupling constants evaluated in this work. Additionally, all geometries of the two test sets as well as the ORCA inputs for the 1-adamantyl timings are provided (PDF)
