# Supplementary material for: A Benchmark and Basis-Set Extrapolation Study of Hyperfine Coupling Constants from the Random Phase Approximation and σ‑Functionals
Source: J Phys Chem A. 2026 Apr 23;130(18):3695–709. doi: 10.1021/acs.jpca.6c00623 (PMC13158915; doi:10.1021/acs.jpca.6c00623)
Supplement: Supplementary file 1 [file jp6c00623_si_001.pdf]

Supporting Information: A Benchmark and Basis-Set Extrapolation Study of Hyperfine Coupling Constants from the Random Phase Approximation and σ-Functionals

Daniel Graf,[*,†,¶] Lu Liu,[†,¶] Florian Siekmann,[†] Viktoria Drontschenko,[†] and Christian Ochsenfeld[*,†,‡]

†*Theoretical Chemistry, Department of Chemistry, Ludwig-Maximilians-Universität München (LMU), D-81377 Munich, Germany*

‡*Max Planck Institute for Solid State Research, D-70569 Stuttgart, Germany*

¶*Contributed equally to this work*

E-mail: daniel.graf@lmu.de; christian.ochsenfeld@uni-muenchen.de

Table S1: Isotropic HFCCs for small main-group compounds using the pcJ-1 basis set.

Radical	Nuclei	Experiment	CBS CCSD(T)	RPA@TPSS	RPA@TPSSh	RPA@TPSS0	RPA@PBE	RPA@PBE0	RPA@B3LYP	RPA@ωB97X-V	σ(W1)@TPSS	σ(W1)@PBE	σ(W1)@PBE0	σ(W1)@B3LYP	σ(S1)@PBE	σ(S1)@PBE0	σ(S2)@PBE	σ(S2)@PBE0	B3LYP	DLPNO-CCSD
BO	^{11}B	1024.9	1031.1	976.5	1004.6	1024.3	966.8	1016.2	1005.6	1065.6	989.7	979.0	1000.7	994.1	988.4	1001.8	989.3	1002.2	1088.3	1017.8
	^{17}O	14.0	-14.7	-2.8	-4.7	-4.5	-2.2	-3.6	-4.8	-8.1	-3.9	-2.3	-2.7	-4.1	-4.6	-2.6	-4.6	-2.7	-9.2	-4.0
BeF	^{9}Be	294.0	-286.2	-287.8	-291.4	-293.8	-289.0	-293.9	-293.6	-298.1	-297.1	-299.4	-297.6	-298.4	-299.0	-296.6	-297.8	-296.0	-313.3	-293.1
	^{19}F	229.0	218.3	306.2	304.4	300.5	299.0	297.0	304.3	290.3	312.8	310.2	308.2	313.4	328.3	310.6	329.6	311.1	338.3	265.6
BeH	^{9}Be	199.3	-193.6	-186.1	-190.8	-194.3	-184.4	-191.8	-191.1	-200.2	-199.2	-196.4	-195.8	-196.8	-196.5	-194.8	-194.4	-194.6	-206.3	-188.9
	^{1}H	193.9	197.9	250.3	240.1	232.4	249.7	237.9	240.8	221.3	242.0	249.3	238.9	242.6	252.7	239.3	253.5	238.8	236.6	242.8
CH	^{1}H	57.7	-56.9	-94.7	-89.3	-83.7	-61.0	-62.2	-62.2	-64.6	-91.7	-60.3	-59.5	-59.6	-58.2	-58.9	-58.2	-59.2	-47.3	-63.7
	^{13}C	47.1	42.3	20.7	20.5	23.5	51.2	41.6	43.4	41.4	36.4	70.3	52.3	58.1	66.5	53.0	64.8	52.4	54.0	23.6
CO^+	^{13}C	1573.0	1538.0	1421.4	1460.2	1506.4	1400.1	1500.0	1474.9	1591.3	1428.3	1408.5	1455.9	1435.8	1418.8	1460.4	1423.9	1462.0	1611.9	1516.2
	^{17}O	18.5	19.2	28.1	30.8	32.3	28.8	32.9	31.4	27.3	29.1	31.6	34.7	32.9	29.5	34.2	29.5	33.7	28.2	33.9
Cl_2^-	^{35}Cl	109.0	125.3	137.4	140.6	139.6	138.6	140.9	137.6	140.1	130.9	135.2	138.1	134.4	135.1	137.5	133.5	137.5	102.9	125.9
OH	^{17}O	51.3	-51.7	-59.1	-56.3	-53.6	-63.6	-56.4	-57.3	-54.8	-67.0	-74.7	-61.8	-66.2	-66.1	-59.1	-62.7	-57.7	-42.0	-43.8
	^{1}H	71.5	-72.2	-71.0	-75.4	-78.6	-66.9	-77.0	-76.1	-81.0	-68.0	-65.1	-71.9	-71.3	-62.7	-71.1	-62.8	-71.5	-68.0	-81.9
SH	^{1}H	65.0	-51.4	-50.3	-53.0	-54.5	-46.2	-52.2	-51.8	-54.2	-49.7	-47.3	-53.0	-52.3	-46.0	-52.5	-45.9	-52.4	-39.8	-59.4
	^{33}S		36.9	57.2	54.5	52.2	58.9	55.0	56.2	51.3	55.3	60.3	55.5	58.7	57.6	55.4	58.1	55.3	27.6	22.1
BH_2	^{11}B	357.9	348.4	377.6	379.9	381.0	347.5	363.1	361.1	376.7	397.6	372.0	371.9	372.2	374.5	372.5	372.7	372.4	374.7	352.7
	^{1}H	38.0	35.5	13.4	14.8	16.8	39.0	33.8	35.0	28.4	16.6	40.8	35.3	37.1	40.0	34.4	38.7	34.2	40.8	27.7
BeOH	^{9}Be	264.0	-259.3	-244.9	-257.6	-259.6	-255.2	-259.6	-259.5	-263.0	-298.5	-265.5	-263.8	-265.2	-265.2	-263.1	-263.9	-262.4	-277.3	-258.0
	^{17}O		-46.1	-63.6	-54.0	-53.2	-53.4	-52.6	-53.4	-50.4	-39.0	-54.0	-54.5	-54.9	-56.4	-55.2	-56.8	-55.3	-61.5	-50.2
	^{1}H	< 5.0	2.4	4.7	2.5	2.3	2.9	2.5	2.4	1.9	-3.3	2.9	2.6	2.6	2.9	2.7	2.9	2.7	3.2	2.0
CH_2	^{13}C		236.6	247.3	249.3	252.7	242.0	249.2	247.9	257.4	268.7	267.0	257.1	260.2	265.4	255.7	263.1	255.0	248.9	244.5
	^{1}H	20.2	-20.1	-29.4	-29.6	-30.5	-18.5	-23.4	-22.6	-28.1	-30.6	-19.2	-20.9	-20.8	-19.0	-20.4	-19.6	-20.7	-11.2	-28.8
CH_2^-	^{13}C	58.9	63.1	29.9	31.1	35.9	44.3	41.2	40.8	48.2	48.7	66.7	52.0	54.5	65.9	51.9	64.4	51.3	67.3	32.9
	^{1}H	44.8	-42.7	-56.8	-57.5	-58.7	-51.4	-55.4	-54.8	-58.5	-56.8	-52.1	-53.5	-53.2	-50.5	-52.9	-50.7	-53.1	-43.2	-56.0
C_2H	^{13}C	213.0	230.5	228.3	228.4	227.9	226.3	224.5	223.8	215.2	233.0	232.4	235.7	232.9	240.4	235.4	237.6	235.4	221.9	214.8
	^{13}C	1014.5	1025.0	970.1	998.3	1017.6	957.9	1015.6	1007.7	1064.2	993.1	989.6	1011.0	1009.9	998.1	1012.4	998.4	1012.2	1078.5	1016.7
	^{1}H	50.4	48.3	53.7	57.9	60.2	53.4	60.7	60.6	65.3	58.7	55.8	58.2	60.8	56.5	57.2	54.5	57.1	60.0	59.4
HCO	^{1}H	377.5	380.6	382.6	386.2	390.4	380.2	389.5	388.2	398.5	399.0	397.2	395.2	396.0	399.4	395.9	397.9	395.5	402.7	384.6
	^{1}H	354.0	359.4	354.3	356.8	358.0	358.4	363.1	362.5	366.5	365.6	367.6	363.2	365.1	363.3	361.9	360.2	361.3	370.5	361.1
	^{17}O	42.3	-44.5	-46.0	-46.5	-47.4	-48.2	-48.2	-49.1	-50.1	-49.6	-52.6	-49.6	-51.8	-50.5	-48.8	-49.3	-48.4	-34.0	-46.5
HCS	^{13}C		278.5	291.8	296.6	300.6	292.9	303.7	303.7	317.8	305.7	307.4	303.3	307.4	306.0	303.5	304.5	303.1	309.5	301.4
	^{1}H	127.5	129.1	121.8	122.1	122.7	126.6	126.4	125.8	122.4	125.9	131.2	129.9	129.2	130.5	129.0	129.0	128.6	133.8	117.3
	^{33}S		22.4	26.1	24.5	24.0	27.4	24.1	25.5	21.9	27.6	29.4	26.1	27.3	30.1	26.1	30.1	26.1	14.2	13.0
HOO	^{1}H	27.4	-23.9	-26.6	-26.6	-25.8	-23.8	-25.2	-25.5	-23.9	-26.4	-20.0	-24.5	-24.6	-22.8	-24.3	-22.8	-24.3	-21.9	-20.9
	^{17}O1		-59.6	-60.4	-61.4	-62.4	-62.8	-63.6	-63.5	-65.5	-65.8	-70.2	-65.5	-68.0	-63.7	-63.5	-61.1	-62.3	-41.6	-62.0
	^{17}O2		-34.8	-36.7	-36.3	-36.2	-40.2	-37.4	-38.4	-39.1	-41.2	-46.2	-39.0	-41.0	-42.1	-37.8	-41.0	-37.5	-26.7	-32.7
H_2O^+	^{17}O	83.2	-79.8	-94.9	-91.7	-91.7	-104.7	-94.8	-96.5	-95.5	-103.3	-113.8	-97.0	-102.9	-103.5	-94.3	-100.3	-93.0	-70.0	-88.4
	^{1}H	73.1	-75.2	-76.5	-80.2	-83.4	-74.3	-83.2	-82.0	-87.1	-74.3	-74.2	-77.8	-77.2	-71.0	-77.1	-71.3	-77.5	-73.0	-86.5
NH_2	^{1}H	67.2	-65.9	-72.5	-74.5	-76.1	-67.8	-73.8	-72.9	-77.8	-70.9	-67.5	-69.8	-69.4	-64.7	-68.9	-64.8	-69.2	-62.7	-76.7
	^{14}N	27.9	27.9	26.6	26.2	26.7	30.8	28.2	28.6	29.4	32.6	38.3	30.9	33.2	35.9	30.2	34.7	29.8	28.1	25.8
CH_3	^{13}C	75.7	74.0	73.3	76.7	81.8	80.3	86.9	85.8	94.4	90.9	100.6	93.6	95.8	97.2	92.8	95.2	92.3	90.4	82.3
	^{1}H	70.1	-69.4	-78.2	-80.0	-82.1	-73.7	-80.0	-79.0	-85.5	-81.6	-75.1	-76.7	-77.2	-74.3	-75.6	-74.1	-75.9	-66.3	-81.6
F_2CH	^{13}C	417.0	419.9	416.5	424.3	429.6	414.2	429.8	429.3	445.6	439.4	437.3	431.8	436.6	432.0	428.2	429.0	427.2	410.9	424.2
	^{19}F	236.0	240.3	271.6	268.9	268.5	289.3	276.9	283.1	286.7	289.8	308.1	281.7	293.8	305.3	277.5	301.0	276.3	213.4	253.4
	^{1}H	62.2	61.3	55.9	53.5	53.0	65.3	59.0	59.6	53.8	54.8	63.5	61.3	61.2	62.6	61.6	61.9	61.4	68.5	50.9
H_2CN	^{13}C	81.0	-74.9	-75.8	-78.2	-80.9	-77.2	-83.4	-82.8	-90.5	-77.6	-78.6	-77.5	-79.5	-75.8	-76.8	-75.7	-76.8	-70.5	-84.9
	^{1}H	233.2	216.1	213.2	212.4	210.7	213.8	212.8	212.4	210.7	215.3	215.5	213.8	214.7	213.9	213.2	213.3	213.4	229.7	219.0
	^{14}N	25.8	25.0	27.3	26.9	26.9	29.5	27.9	28.2	28.4	31.0	34.7	29.5	31.4	32.0	28.6	31.1	28.1	23.4	25.4
NH_3^+	^{14}N	54.9	45.5	43.6	45.2	47.5	47.5	49.9	49.4	52.7	49.7	54.2	51.1	52.6	52.4	50.2	51.1	49.9	44.3	50.4
	^{1}H	76.8	-80.7	-83.3	-86.0	-89.0	-80.8	-88.5	-87.7	-94.0	-84.7	-80.8	-83.4	-84.3	-79.6	-82.4	-79.6	-82.9	-77.2	-91.9
PH_3^+	^{31}P	1176.0	1181.2	1218.0	1235.5	1263.9	1258.2	1288.1	1283.7	1331.0	1268.5	1292.7	1269.1	1281.7	1284.2	1262.2	1281.2	1262.8	1136.4	1184.4
	^{1}H	< 5.9	3.0	-0.4	-1.3	-2.6	7.0	2.0	2.9	-2.2	2.4	10.9	5.9	7.2	13.3	6.6	13.0	6.5	13.0	-6.1
CH_2CH	^{13}C	24.0	-12.7	-12.5	-13.8	-16.3	-14.9	-19.8	-18.9	-26.2	-13.6	-15.9	-15.1	-16.2	-13.9	-14.8	-14.1	-14.8	-14.0	-23.5
	^{13}C	301.5	311.0	317.4	321.9	329.2	318.5	330.8	328.9	345.9	335.9	338.4	331.4	334.6	334.9	330.5	332.8	329.9	331.6	326.5
	^{1}H	192.0	163.0	164.5	164.1	162.6	165.2	164.8	164.5	163.2	168.4	168.9	165.8	167.8	167.1	164.7	166.3	164.6	178.4	165.8
	^{1}H	95.8	97.0	95.7	95.5	95.2	94.6	95.7	94.9	95.9	95.3	93.4	93.5	94.2	93.2	93.0	93.3	93.3	109.6	100.3
	^{1}H	37.3	43.8	34.8	33.9	31.6	42.0	36.4	37.2	30.1	33.4	41.1	40.7	40.0	41.5	40.8	40.8	40.5	51.4	29.3
H_2CCN	^{13}CCH2		67.2	68.9	72.7	77.3	72.4	80.2	79.3	89.5	82.3	86.3	81.5	84.2	83.5	80.4	81.8	79.8	75.9	79.8
	^{13}CCN		-59.7	-65.9	-69.6	-73.5	-65.7	-75.1	-73.8	-85.2	-65.5	-62.8	-63.8	-65.8	-60.0	-63.3	-59.7	-63.3	-62.4	-77.9
	^{1}H	58.8	-59.8	-67.6	-70.4	-73.2	-62.5	-71.0	-69.5	-77.7	-70.9	-63.6	-66.1	-66.3	-62.4	-65.2	-62.1	-65.5	-59.1	-73.5
	^{14}N	9.8	7.8	9.8	9.7	9.9	10.6	10.4	10.2	11.2	11.3	13.0	10.8	11.4	11.8	10.3	11.4	10.1	9.4	9.8
H_2CCO^+	^{13}CCH2		63.8	63.0	65.8	69.6	67.0	72.8	72.2	79.5	75.6	80.9	75.5	78.1	77.4	74.6	75.5	74.1	74.6	72.5
	^{13}CCO		-56.9	-59.2	-62.2	-65.5	-59.4	-67.5	-66.5	-74.2	-56.2	-52.4	-56.4	-57.6	-50.4	-55.9	-50.5	-56.1	-52.8	-72.7
	^{1}H	58.0	-60.2	-65.3	-67.6	-70.3	-61.0	-69.2	-68.0	-74.9	-69.1	-62.0	-65.2	-65.3	-60.5	-64.4	-60.3	-64.6	-59.3	-72.2
	^{17}O		-15.3	-21.8	-22.2	-22.9	-23.4	-23.6	-23.7	-25.0	-23.5	-25.4	-23.4	-24.7	-23.1	-22.6	-22.3	-22.2	-15.3	-22.0
MAE to CCSD(T) [MHz]				13.42	12.31	12.19	12.95	11.79	12.32	16.60	16.65	17.08	12.53	14.44	15.69	11.79	14.67	11.57	14.58	10.20
MAPE to CCSD(T) [%]				17.98	17.09	18.88	14.64	14.71	13.87	20.94	21.25	22.49	14.93	17.45	20.71	14.40	19.44	13.98	18.60	21.77
MAE to exp. [MHz]				17.95	16.31	15.46	17.36	14.43	15.11	17.70	20.15	20.02	15.54	17.07	18.96	14.90	18.02	14.74	15.19	11.68
MAPE to exp. [%]				16.94	16.81	16.88	12.80	13.25	12.71	15.18	18.01	16.89	13.36	13.98	14.95	12.73	13.91	12.48	13.22	15.03

Table S2: Isotropic HFCCs for small main-group compounds using the pcJ-2 basis set.

Radical	Nuclei	Experiment	CBS CCSD(T)	RPA@TPSS	RPA@TPSSh	RPA@TPSS0	RPA@PBE	RPA@PBE0	RPA@B3LYP	RPA@ωB97X-V	σ(W1)@TPSS	σ(W1)@PBE	σ(W1)@PBE0	σ(W1)@B3LYP	σ(S1)@PBE	σ(S1)@PBE0	σ(S2)@PBE	σ(S2)@PBE0	B3LYP	DLPNO-CCSD
BO	^{11}B	1024.9	1031.1	979.1	1003.8	1026.5	970.8	1019.6	1010.5	1071.5	996.8	988.8	1007.0	1001.6	989.3	1010.7	996.5	1012.0	1083.3	1027.8
	^{17}O	14.0	-14.7	-2.5	-3.7	-4.1	-2.0	-3.4	-4.2	-7.6	-3.6	-2.1	-2.5	-3.3	-4.6	-3.0	-3.7	-3.2	-9.3	-5.4
BeF	^{9}Be	294.0	-286.2	-291.0	-295.0	-297.4	-293.6	-298.0	-297.4	-301.2	-304.9	-308.8	-305.2	-306.3	-309.6	-304.1	-307.5	-303.4	-312.5	-294.5
	^{19}F	229.0	218.3	230.9	229.0	224.7	225.5	223.2	228.2	218.5	238.1	227.7	223.5	228.7	232.0	222.0	231.0	221.9	277.5	218.4
BeH	^{9}Be	199.3	-193.6	-196.0	-204.9	-209.0	-197.7	-205.3	-204.3	-213.4	-205.5	-205.5	-207.5	-207.4	-207.4	-207.8	-206.5	-208.0	-208.0	-197.8
	^{1}H	193.9	197.9	210.2	191.6	182.8	208.8	194.1	196.5	178.3	222.7	227.0	202.4	208.2	219.4	199.9	216.6	198.2	215.3	201.5
CH	^{1}H	57.7	-56.9	-94.3	-90.2	-84.1	-56.2	-58.3	-57.4	-61.3	-94.8	-57.2	-57.7	-57.0	-58.2	-57.5	-55.7	-57.3	-47.2	-61.3
	^{13}C	47.1	42.3	38.7	29.8	33.5	64.9	51.3	52.4	51.0	46.5	75.9	58.5	62.1	64.8	57.5	71.5	56.6	54.8	28.9
CO^+	^{13}C	1573.0	1538.0	1433.4	1465.4	1517.6	1411.9	1509.1	1485.1	1598.9	1467.9	1454.0	1483.7	1468.0	1423.9	1490.5	1460.1	1490.6	1595.8	1524.6
	^{17}O	18.5	19.2	29.5	33.5	32.6	30.6	33.9	33.0	29.1	30.1	32.4	34.7	33.6	29.5	34.2	30.9	33.8	30.4	33.7
Cl_2^-	^{35}Cl	109.0	125.3	125.5	125.3	126.4	122.6	127.3	128.1	126.0	121.1	119.0	124.6	125.5	133.5	122.4	116.7	122.3	98.6	131.8
OH	^{17}O	51.3	-51.7	-59.9	-57.1	-56.2	-65.1	-58.5	-57.1	-55.6	-61.1	-70.6	-61.5	-61.8	-62.7	-58.0	-60.2	-56.7	-43.7	-42.2
	^{1}H	71.5	-72.2	-65.6	-69.4	-72.1	-61.4	-70.4	-69.8	-74.8	-65.4	-64.3	-67.2	-66.9	-62.8	-66.6	-60.2	-66.6	-66.1	-77.2
SH	^{1}H	65.0	-51.4	-45.2	-47.2	-48.3	-40.1	-45.4	-44.8	-48.2	-49.2	-44.2	-48.0	-48.0	-45.9	-48.0	-42.7	-47.6	-40.1	-56.6
	^{33}S		36.9	44.2	40.1	40.5	49.6	44.6	47.7	38.5	39.8	48.8	44.7	47.5	58.1	42.8	46.3	43.0	20.9	28.9
BH_2	^{11}B	357.9	348.4	383.0	384.6	384.8	348.8	363.7	362.2	377.1	391.4	356.6	360.8	360.5	372.7	361.6	357.9	362.7	366.1	346.5
	^{1}H	38.0	35.5	15.1	15.8	17.7	41.9	36.0	37.0	29.7	12.8	40.0	36.8	37.2	38.7	36.8	40.8	36.8	42.8	32.0
BeOH	^{9}Be	264.0	-259.3	-265.2	-267.4	-270.7	-267.8	-271.6	-270.8	-273.6	-277.2	-281.4	-278.5	-278.9	-281.8	-277.8	-280.3	-277.2	-282.5	-266.5
	^{17}O		-46.1	-47.0	-47.8	-45.2	-46.4	-45.4	-45.6	-42.6	-47.6	-46.7	-46.0	-46.1	-46.4	-45.9	-46.3	-45.8	-56.4	-44.9
	^{1}H	< 5.0	2.4	2.8	2.9	2.5	2.8	2.6	2.5	2.1	2.7	2.9	2.8	2.7	2.6	2.7	2.6	2.7	3.7	2.2
CH_2	^{13}C		236.6	249.0	251.1	253.6	240.0	248.0	247.1	256.0	260.2	252.5	247.5	249.2	263.1	246.8	250.6	246.9	241.1	234.3
	^{1}H	20.2	-20.1	-27.5	-28.6	-28.7	-13.7	-20.0	-19.2	-25.3	-31.5	-16.2	-17.7	-18.2	-19.6	-16.7	-14.6	-16.6	-10.2	-24.3
CH_2^-	^{13}C	58.9	63.1	42.0	45.0	49.1	51.5	54.0	54.9	61.1	52.4	62.0	54.6	58.4	64.4	54.6	59.9	54.1	73.1	39.7
	^{1}H	44.8	-42.7	-51.4	-51.7	-52.7	-45.0	-49.1	-49.0	-53.0	-54.2	-47.8	-47.9	-48.4	-50.7	-47.5	-45.8	-47.4	-40.9	-51.7
C_2H	^{13}C	213.0	230.5	228.4	230.8	229.5	228.3	226.7	228.0	219.2	224.1	225.2	231.2	229.0	237.6	231.8	229.4	232.2	226.2	221.8
	^{13}C	1014.5	1025.0	926.0	1008.5	1031.9	970.6	1030.6	1016.2	1077.0	1079.0	1000.3	1027.8	1018.0	998.4	1030.7	1006.9	1031.5	1075.0	1025.8
	^{1}H	50.4	48.3	53.3	55.8	57.5	50.9	57.7	58.0	62.5	48.1	52.5	55.0	57.5	54.5	53.7	50.4	53.6	58.6	56.6
HCO	^{1}H	377.5	380.6	378.8	383.7	388.5	375.4	386.5	385.6	398.1	384.8	381.8	383.3	383.6	397.9	385.3	381.9	385.8	394.9	378.4
	^{1}H	354.0	359.4	350.7	352.7	354.7	356.8	361.1	359.8	364.4	355.8	362.2	360.9	359.7	360.2	361.0	358.3	361.0	378.0	362.1
	^{17}O	42.3	-44.5	-45.2	-45.4	-46.2	-46.8	-46.9	-47.5	-48.3	-47.7	-49.1	-47.0	-48.6	-49.3	-45.8	-46.3	-45.5	-35.4	-42.6
HCS	^{13}C		278.5	283.8	288.7	293.7	284.6	295.8	295.1	310.6	289.3	289.5	289.2	291.2	304.5	290.5	289.0	290.9	299.0	287.7
	^{1}H	127.5	129.1	120.8	120.9	121.1	126.4	126.0	125.7	122.0	122.1	128.2	128.9	127.6	129.0	128.9	127.7	128.8	135.7	119.8
	^{33}S		22.4	23.4	21.9	21.3	23.9	20.9	22.4	19.1	23.3	22.4	21.0	22.5	30.1	20.9	22.1	21.0	13.6	17.0
HOO	^{1}H	27.4	-23.9	-24.8	-25.1	-24.2	-22.5	-23.3	-23.6	-22.2	-24.6	-22.3	-23.3	-23.4	-22.8	-23.1	-21.8	-23.2	-21.5	-19.4
	^{17}O1		-59.6	-63.0	-62.7	-64.8	-65.0	-65.4	-65.8	-66.5	-65.7	-66.7	-64.5	-66.9	-61.1	-62.4	-58.9	-61.5	-44.4	-58.8
	^{17}O2		-34.8	-36.2	-35.8	-35.7	-39.5	-36.9	-38.0	-38.5	-40.3	-42.2	-36.6	-39.0	-41.0	-35.4	-38.7	-35.0	-27.5	-29.5
H_2O^+	^{17}O	83.2	-79.8	-86.7	-84.9	-84.2	-96.5	-87.6	-89.6	-87.7	-89.7	-103.0	-89.2	-93.7	-100.3	-85.7	-91.2	-84.5	-65.3	-75.5
	^{1}H	73.1	-75.2	-70.2	-72.9	-76.1	-68.0	-75.8	-74.6	-79.9	-70.3	-69.4	-71.5	-70.6	-71.3	-70.8	-66.1	-70.9	-70.7	-80.3
NH_2	^{1}H	67.2	-65.9	-66.9	-69.0	-70.3	-61.1	-67.4	-66.2	-71.8	-68.5	-63.7	-64.9	-64.4	-64.8	-64.2	-60.4	-64.2	-60.5	-71.9
	^{14}N	27.9	27.9	28.5	28.4	28.9	31.8	30.4	30.8	31.3	31.3	35.9	31.4	32.9	34.7	30.3	33.3	29.8	28.4	24.7
CH_3	^{13}C	75.7	74.0	73.2	76.3	80.4	79.0	84.9	84.2	91.9	85.6	92.2	85.9	87.8	95.2	84.5	87.1	83.7	83.1	71.0
	^{1}H	70.1	-69.4	-73.2	-75.7	-77.4	-66.9	-74.5	-73.4	-80.7	-77.5	-68.8	-70.9	-71.0	-74.1	-69.9	-65.8	-69.9	-64.8	-76.7
F_2CH	^{13}C	417.0	419.9	418.5	425.1	432.7	417.5	433.2	432.1	448.9	435.0	433.2	429.3	433.0	429.0	428.1	428.2	428.1	409.8	420.6
	^{19}F	236.0	240.3	253.3	253.2	253.5	269.4	260.7	265.8	269.6	265.3	277.7	258.5	268.0	301.0	252.6	264.3	251.7	218.7	235.8
	^{1}H	62.2	61.3	56.2	54.6	53.4	66.4	60.7	61.6	55.1	53.1	63.5	63.0	62.8	61.9	63.7	63.1	63.7	71.0	55.0
H_2CN	^{13}C	81.0	-74.9	-72.6	-75.2	-77.7	-73.7	-79.8	-79.4	-86.9	-76.3	-78.0	-77.6	-78.7	-75.7	-77.0	-75.4	-77.0	-67.9	-81.5
	^{1}H	233.2	216.1	213.3	211.6	209.2	214.2	211.8	211.2	209.3	219.0	220.6	217.8	217.7	213.3	217.3	218.5	217.1	231.7	218.5
	^{14}N	25.8	25.0	28.8	28.3	28.3	30.5	29.1	29.4	29.6	30.7	34.2	30.4	31.7	31.1	29.8	31.7	29.4	23.7	24.8
NH_3^+	^{14}N	54.9	45.5	41.9	43.5	45.4	46.0	48.0	47.8	50.8	46.2	50.7	47.8	49.1	51.1	46.6	46.9	46.2	41.8	44.4
	^{1}H	76.8	-80.7	-78.8	-80.7	-83.8	-75.7	-83.3	-82.2	-88.8	-80.5	-76.2	-78.8	-78.9	-79.6	-77.5	-73.3	-77.7	-75.7	-86.6
PH_3^+	^{31}P	1176.0	1181.2	1178.8	1200.2	1226.7	1213.9	1256.4	1253.9	1301.2	1200.4	1212.9	1216.7	1224.4	1281.2	1213.1	1201.4	1217.5	1104.4	1211.3
	^{1}H	< 5.9	3.0	4.5	3.9	3.0	14.4	8.2	8.8	3.6	1.4	12.0	9.1	9.1	13.0	8.6	13.8	9.0	14.0	-0.3
CH_2CH	^{13}C	24.0	-12.7	-10.6	-12.4	-14.2	-13.5	-17.9	-16.9	-23.8	-14.5	-17.7	-16.0	-17.0	-14.1	-15.4	-15.6	-15.3	-11.7	-19.9
	^{13}C	301.5	311.0	315.7	321.4	328.0	316.6	329.1	327.3	344.0	327.0	329.3	325.3	326.9	332.8	325.4	325.5	325.5	322.8	317.4
	^{1}H	192.0	163.0	163.5	162.3	160.6	164.1	162.9	162.7	161.3	168.5	170.1	166.8	168.2	166.3	166.1	168.7	165.9	178.7	165.3
	^{1}H	95.8	97.0	95.8	95.3	94.5	94.6	95.0	94.2	94.9	98.9	97.8	97.1	97.3	93.3	96.9	98.0	96.8	110.7	100.5
	^{1}H	37.3	43.8	38.1	36.5	35.0	45.9	40.8	41.3	34.0	35.0	45.1	46.1	44.7	40.8	46.7	46.0	46.6	55.1	35.9
H_2CCN	^{13}CCH2		67.2	68.6	72.0	76.3	71.6	79.1	78.4	88.2	79.2	83.1	78.0	80.9	81.8	77.3	77.8	76.7	71.2	72.3
	^{13}CCN		-59.7	-61.8	-65.5	-69.6	-62.2	-71.5	-69.8	-81.6	-62.5	-61.9	-64.7	-65.2	-59.7	-64.3	-60.3	-64.5	-59.9	-73.9
	^{1}H	58.8	-59.8	-64.1	-66.8	-69.6	-58.2	-66.5	-64.7	-73.9	-68.6	-60.2	-61.8	-61.6	-62.1	-61.1	-58.0	-61.1	-58.4	-69.2
	^{14}N	9.8	7.8	10.6	10.5	10.6	11.3	11.0	11.0	11.8	11.5	13.1	11.3	11.8	11.4	10.9	12.0	10.7	9.7	9.5
H_2CCO^+	^{13}CCH2		63.8	63.1	65.4	68.6	66.5	71.6	71.4	77.8	72.8	77.4	73.0	75.2	75.5	72.3	72.1	71.9	69.0	65.8
	^{13}CCO		-56.9	-55.5	-58.8	-61.7	-57.9	-64.1	-63.3	-71.7	-53.3	-54.2	-56.4	-57.1	-50.5	-56.2	-53.4	-56.7	-52.3	-69.0
	^{1}H	58.0	-60.2	-62.8	-65.1	-67.0	-58.2	-64.6	-63.6	-71.3	-66.8	-59.9	-60.3	-60.6	-60.3	-59.7	-57.6	-59.7	-58.7	-68.1
	^{17}O		-15.3	-20.9	-21.1	-21.5	-21.9	-22.2	-22.4	-23.4	-22.2	-23.4	-21.7	-22.9	-22.3	-20.9	-20.8	-20.6	-15.0	-19.1
MAE to CCSD(T) [MHz]				9.56	8.12	8.17	9.48	7.66	8.06	13.63	11.11	10.81	7.17	8.62	13.14	6.56	8.92	6.46	13.23	6.07
MAPE to CCSD(T) [%]				11.92	11.79	11.72	17.03	13.14	13.15	15.28	14.48	19.03	13.37	14.60	18.26	12.20	17.10	11.96	19.47	13.24
MAE to exp. [MHz]				13.46	11.28	11.49	13.04	10.16	10.35	15.07	14.19	12.89	10.23	11.00	16.08	9.63	11.21	9.59	14.16	8.92
MAPE to exp. [%]				13.85	13.82	13.89	12.71	10.36	10.19	12.48	15.07	14.01	11.23	11.56	12.97	10.62	12.45	10.35	13.84	11.76

Table S3: Isotropic HFCCs for small main-group compounds using the pcJ-3 basis set.

Radical	Nuclei	Experiment	CBS CCSD(T)	RPA@TPSS	RPA@TPSSh	RPA@TPSS0	RPA@PBE	RPA@PBE0	RPA@B3LYP	RPA@ωB97X-V	σ(W1)@TPSS	σ(W1)@PBE	σ(W1)@PBE0	σ(W1)@B3LYP	σ(S1)@PBE	σ(S1)@PBE0	σ(S2)@PBE	σ(S2)@PBE0	B3LYP	DLPNO-CCSD
BO	^{11}B	1024.9	1031.1	998.8	1017.0	1042.7	985.5	1034.1	1025.8	1086.8	1014.3	1001.8	1021.5	1016.4	1007.1	1027.7	1012.1	1029.7	1089.0	1042.5
	^{17}O	14.0	-14.7	-3.8	-3.9	-4.8	-2.2	-3.8	-4.7	-7.8	-4.8	-2.4	-3.0	-4.0	-4.3	-3.7	-4.2	-4.0	-9.6	-4.4
BeF	^{9}Be	294.0	-286.2	-277.3	-282.3	-285.5	-282.3	-287.0	-286.9	-290.9	-275.7	-282.8	-286.1	-285.0	-283.4	-287.3	-286.0	-287.5	-306.0	-286.3
	^{19}F	229.0	218.3	209.0	206.6	203.4	201.6	202.0	205.2	198.3	215.5	205.1	202.2	206.5	210.8	201.6	211.0	201.4	260.9	206.4
BeH	^{9}Be	199.3	-193.6	-194.8	-198.9	-203.2	-192.4	-200.2	-199.8	-208.5	-193.0	-191.5	-197.3	-196.0	-193.0	-198.2	-194.5	-198.4	-206.5	-194.4
	^{1}H	193.9	197.9	182.2	176.8	169.0	195.0	183.9	183.5	168.6	185.5	204.9	190.3	191.1	203.7	190.6	206.2	189.7	209.7	193.0
CH	^{1}H	57.7	-56.9	-97.9	-92.2	-86.1	-55.9	-58.5	-58.3	-61.6	-98.4	-56.9	-58.1	-58.3	-54.8	-58.1	-55.0	-57.9	-47.7	-59.7
	^{13}C	47.1	42.3	34.1	34.3	37.6	70.1	57.2	58.7	55.3	42.3	81.4	63.1	66.9	76.6	61.8	75.1	60.9	56.1	35.7
CO^+	^{13}C	1573.0	1538.0	1443.9	1491.6	1534.6	1439.1	1528.9	1509.1	1621.2	1491.1	1490.6	1509.5	1500.8	1489.2	1518.6	1492.0	1519.9	1603.4	1545.6
	^{17}O	18.5	19.2	32.7	31.5	33.4	29.1	34.3	31.9	29.1	31.8	30.1	34.6	31.6	28.1	33.8	28.5	33.5	30.7	34.9
Cl_2^-	^{35}Cl	109.0	125.3	122.5	122.1	122.0	123.2	123.0	124.3	120.7	118.8	120.3	121.9	123.5	118.7	121.3	119.9	121.3	96.3	124.6
OH	^{17}O	51.3	-51.7	-63.1	-60.0	-58.3	-71.4	-61.1	-64.2	-58.3	-66.2	-79.9	-65.4	-71.3	-68.5	-62.0	-66.5	-60.5	-45.3	-46.4
	^{1}H	71.5	-72.2	-65.3	-69.0	-71.8	-61.0	-70.3	-69.1	-74.7	-65.4	-61.8	-67.2	-66.2	-58.8	-66.7	-58.9	-66.6	-66.5	-75.2
SH	^{1}H	65.0	-51.4	-45.7	-47.6	-48.7	-40.0	-45.2	-44.7	-47.9	-50.6	-44.9	-47.4	-47.5	-42.5	-47.0	-42.7	-46.7	-40.3	-53.0
	^{33}S		36.9	45.3	42.2	37.9	50.4	42.6	44.6	35.3	40.6	45.7	42.0	44.1	43.1	41.3	44.2	41.3	18.0	25.7
BH_2	^{11}B	357.9	348.4	385.2	386.4	386.7	347.3	362.6	361.5	377.6	389.3	351.1	356.0	355.5	350.8	356.6	352.8	357.2	365.9	348.2
	^{1}H	38.0	35.5	12.7	13.9	15.9	40.4	35.3	35.7	29.0	11.0	37.4	35.1	34.8	37.4	34.7	37.4	34.6	43.4	32.5
BeOH	^{9}Be	264.0	-259.3	-254.2	-257.5	-260.3	-257.5	-261.5	-261.5	-264.9	-252.3	-257.3	-260.4	-259.4	-257.3	-261.7	-260.1	-261.9	-277.7	-259.1
	^{17}O		-46.1	-44.5	-43.5	-42.6	-43.9	-42.5	-43.0	-40.3	-44.2	-43.7	-43.1	-43.3	-44.7	-43.4	-44.9	-43.4	-54.9	-44.2
	^{1}H	< 5.0	2.4	2.6	2.4	2.4	2.8	2.6	2.5	2.2	2.3	2.7	2.6	2.6	2.6	2.6	2.7	2.6	3.8	2.3
CH_2	^{13}C		236.6	252.4	253.8	255.8	241.1	248.4	247.4	256.9	261.3	249.5	244.2	246.3	245.8	243.7	245.8	243.6	240.9	235.4
	^{1}H	20.2	-20.1	-28.8	-29.0	-29.3	-14.0	-19.7	-18.8	-25.4	-33.3	-17.2	-17.7	-18.3	-15.5	-16.9	-15.2	-16.9	-10.1	-23.5
CH_2^-	^{13}C	58.9	63.1	51.7	56.0	60.7	62.3	66.6	66.1	73.2	60.6	70.1	66.2	68.4	66.7	65.1	65.9	64.8	81.8	53.4
	^{1}H	44.8	-42.7	-48.7	-49.8	-50.8	-42.8	-47.2	-46.6	-51.1	-51.8	-45.9	-46.3	-46.2	-44.1	-46.2	-44.1	-46.1	-39.5	-47.9
C_2H	^{13}C	213.0	230.5	229.5	230.9	229.0	227.3	226.1	226.0	218.1	222.7	221.2	228.8	223.9	224.2	229.6	225.0	229.8	227.8	220.9
	^{13}C	1014.5	1025.0	993.9	1019.8	1044.3	983.7	1041.8	1033.7	1089.8	1013.1	1009.4	1035.7	1032.6	1008.7	1037.6	1010.7	1038.4	1080.0	1038.4
	^{1}H	50.4	48.3	51.2	54.9	56.4	50.8	56.9	57.1	61.8	52.0	50.0	53.0	55.0	49.2	52.1	49.2	51.9	58.7	55.6
HCO	^{1}H	377.5	380.6	377.0	382.3	388.0	373.6	386.0	384.1	398.4	381.7	377.8	380.3	379.6	375.2	381.3	376.1	381.7	393.8	379.4
	^{1}H	354.0	359.4	349.2	351.3	353.2	355.7	359.6	358.3	363.4	350.4	358.3	358.0	356.2	356.9	358.5	357.2	358.4	379.4	362.2
	^{17}O	42.3	-44.5	-45.5	-45.7	-46.3	-47.3	-47.1	-48.2	-48.4	-48.7	-50.3	-47.6	-49.8	-47.8	-46.6	-46.9	-46.1	-35.8	-42.0
HCS	^{13}C		278.5	283.6	288.9	293.6	276.4	296.1	295.0	311.2	289.7	283.7	289.0	290.8	301.4	289.1	308.5	289.2	295.3	288.9
	^{1}H	127.5	129.1	119.7	119.1	119.8	133.3	124.6	124.2	120.8	118.7	126.9	126.6	125.0	105.2	126.8	99.1	126.6	135.6	118.6
	^{33}S		22.4	22.9	20.1	19.1	30.5	18.8	20.1	16.4	21.5	13.7	18.6	19.7	6.3	18.6	9.5	18.7	12.0	14.3
HOO	^{1}H	27.4	-23.9	-24.9	-25.1	-24.2	-22.1	-23.3	-23.7	-22.3	-24.8	-21.8	-23.3	-23.5	-21.2	-23.1	-21.2	-23.2	-21.6	-19.2
	^{17}O1		-59.6	-64.9	-64.1	-66.0	-66.2	-67.1	-68.5	-68.6	-69.5	-71.2	-67.8	-71.7	-64.3	-65.5	-62.0	-64.3	-45.7	-60.3
	^{17}O2		-34.8	-36.8	-36.2	-36.1	-40.2	-37.4	-38.6	-38.8	-41.6	-43.9	-37.6	-40.4	-41.4	-36.6	-40.2	-36.2	-27.7	-29.6
H_2O^+	^{17}O	83.2	-79.8	-86.9	-84.8	-84.6	-97.4	-89.0	-90.5	-88.6	-92.9	-106.9	-92.1	-97.1	-97.6	-88.3	-95.0	-86.9	-65.8	-76.2
	^{1}H	73.1	-75.2	-69.5	-72.4	-75.3	-67.8	-75.2	-74.1	-79.5	-69.9	-69.2	-71.0	-70.3	-66.1	-70.4	-66.1	-70.4	-70.8	-78.3
NH_2	^{1}H	67.2	-65.9	-66.1	-67.5	-69.4	-60.4	-66.8	-65.9	-71.5	-68.0	-63.2	-64.4	-64.1	-60.2	-63.9	-60.2	-63.8	-60.7	-69.5
	^{14}N	27.9	27.9	29.5	29.4	30.0	33.9	31.9	32.3	32.6	33.4	38.5	32.9	34.8	35.6	31.9	34.9	31.4	29.0	26.2
CH_3	^{13}C	75.7	74.0	73.6	76.4	80.4	80.2	85.5	84.9	92.8	85.8	92.2	84.4	87.3	87.1	84.0	86.0	83.2	83.0	73.7
	^{1}H	70.1	-69.4	-72.9	-74.3	-76.5	-66.3	-73.3	-72.0	-80.1	-76.8	-68.4	-69.7	-69.5	-65.8	-69.1	-65.6	-69.0	-64.9	-74.3
F_2CH	^{13}C	417.0	419.9	421.5	426.6	434.5	419.3	433.7	432.8	450.8	434.8	431.3	427.5	430.9	426.8	426.4	426.7	426.2	410.8	423.4
	^{19}F	236.0	240.3	249.9	249.9	250.6	265.0	258.3	262.7	266.1	264.5	275.8	257.6	266.9	264.8	251.7	261.6	250.2	219.7	233.9
	^{1}H	62.2	61.3	53.9	53.6	52.6	65.4	60.8	61.5	54.9	49.2	61.0	62.5	61.6	61.5	63.3	61.7	63.3	71.5	55.7
H_2CN	^{13}C	81.0	-74.9	-72.7	-75.2	-77.2	-74.5	-80.1	-79.0	-87.0	-77.0	-78.9	-78.2	-78.5	-76.5	-77.8	-76.4	-77.7	-68.1	-81.7
	^{1}H	233.2	216.1	215.0	213.1	210.9	214.8	213.0	213.3	210.5	220.9	222.0	219.9	220.9	221.7	220.1	221.1	220.1	232.4	219.8
	^{14}N	25.8	25.0	29.4	29.0	29.1	31.2	30.2	30.7	30.5	32.2	35.7	31.7	33.5	32.8	30.9	32.1	30.4	23.9	25.5
NH_3^+	^{14}N	54.9	45.5	41.9	43.2	45.4	46.4	48.4	48.2	51.2	46.5	51.7	48.4	49.8	48.2	47.4	47.3	47.0	42.0	45.1
	^{1}H	76.8	-80.7	-76.7	-79.7	-82.6	-75.5	-82.7	-81.5	-88.6	-78.9	-76.5	-78.3	-78.3	-73.6	-77.0	-73.5	-77.2	-76.0	-84.8
PH_3^+	^{31}P	1176.0	1181.2	1130.3	1149.8	1181.8	1178.2	1212.9	1208.1	1259.7	1154.6	1175.9	1169.8	1174.3	1160.2	1162.0	1160.9	1162.9	1080.5	1170.2
	^{1}H	< 5.9	3.0	2.8	2.3	1.7	12.2	7.9	8.3	3.6	-0.9	8.0	6.8	6.7	8.8	6.0	9.0	6.2	14.9	1.5
CH_2CH	^{13}C	24.0	-12.7	-11.1	-12.4	-14.2	-12.8	-18.1	-17.1	-24.3	-16.0	-17.9	-16.9	-17.8	-16.2	-16.4	-15.9	-16.3	-11.6	-20.3
	^{13}C	301.5	311.0	316.3	321.5	328.3	314.8	329.5	327.4	345.3	325.3	324.0	323.2	324.3	319.4	322.5	319.6	322.4	322.7	319.0
	^{1}H	192.0	163.0	164.5	163.3	161.3	166.7	163.9	163.9	162.0	168.7	172.0	168.2	169.5	171.7	167.9	171.5	167.8	179.2	166.1
	^{1}H	95.8	97.0	97.6	96.9	95.8	96.6	96.3	95.7	96.0	102.2	101.5	99.7	100.3	102.2	99.9	101.6	100.0	111.1	101.5
	^{1}H	37.3	43.8	38.2	37.1	35.5	48.9	41.7	42.4	34.5	33.4	46.5	46.3	44.8	47.8	47.1	48.1	47.0	55.9	37.1
H_2CCN	^{13}CCH2		67.2	69.0	72.3	76.4	72.4	79.5	78.9	89.0	79.2	83.0	78.2	80.8	79.3	77.4	78.3	76.8	71.1	73.4
	^{13}CCN		-59.7	-61.8	-65.4	-69.0	-62.5	-71.1	-70.0	-82.1	-62.6	-62.6	-64.6	-65.9	-61.0	-64.4	-60.8	-64.6	-60.0	-73.6
	^{1}H	58.8	-59.8	-63.9	-66.4	-68.7	-57.9	-65.3	-64.2	-73.5	-67.8	-60.1	-60.8	-61.4	-58.2	-60.3	-58.0	-60.3	-58.5	-67.5
	^{14}N	9.8	7.8	10.8	10.8	10.9	11.8	11.6	11.4	12.2	12.2	13.8	11.9	12.4	12.6	11.5	12.4	11.3	9.8	10.1
H_2CCO^+	^{13}CCH2		63.8	63.5	65.8	68.8	67.5	71.9	71.8	78.5	72.0	77.6	72.9	75.1	74.5	72.3	73.7	71.8	68.7	66.5
	^{13}CCO		-56.9	-56.2	-58.4	-61.8	-57.5	-64.5	-63.0	-71.7	-54.3	-53.8	-57.4	-57.3	-53.1	-57.5	-53.1	-57.9	-52.6	-68.4
	^{1}H	58.0	-60.2	-62.9	-64.4	-66.5	-57.4	-64.3	-62.8	-70.7	-66.7	-59.0	-60.2	-59.9	-57.0	-59.7	-56.8	-59.7	-58.7	-66.3
	^{17}O		-15.3	-20.9	-21.3	-21.6	-22.5	-22.5	-22.8	-23.6	-22.9	-24.7	-22.3	-23.8	-22.5	-21.7	-21.8	-21.3	-15.2	-19.7
MAE to CCSD(T) [MHz]				9.08	7.49	7.36	8.17	6.72	6.72	14.32	9.58	8.85	5.90	6.97	8.46	5.47	8.12	5.27	13.46	5.24
MAPE to CCSD(T) [%]				11.65	11.41	12.30	16.61	13.41	13.30	16.42	16.34	17.97	12.66	14.02	16.50	11.47	15.98	11.08	20.16	11.40
MAE to exp. [MHz]				13.23	11.09	11.12	12.13	9.71	9.61	15.70	12.11	11.01	8.71	9.41	10.83	8.32	10.43	8.19	14.50	7.81
MAPE to exp. [%]				14.50	13.59	13.79	13.53	11.01	10.91	13.29	15.65	14.62	11.52	12.19	13.52	10.95	13.28	10.60	14.00	10.11

Table S4: Isotropic HFCCs for small main-group compounds using the pcJ-4 basis set.

Radical	Nuclei	Experiment	CBS CCSD(T)	RPA@TPSS	RPA@TPSSh	RPA@TPSS0	RPA@PBE	RPA@PBE0	RPA@B3LYP	RPA@ωB97X-V	σ(W1)@TPSS	σ(W1)@PBE	σ(W1)@PBE0	σ(W1)@B3LYP	σ(S1)@PBE	σ(S1)@PBE0	σ(S2)@PBE	σ(S2)@PBE0	B3LYP	DLPNO-CCSD
BO	^{11}B	1024.9	1031.1	995.4	1020.2	1043.7	986.7	1035.8	1027.5	1090.5	1001.6	995.7	1018.4	1011.7	1002.0	1024.3	1008.7	1026.7	1089.4	1046.3
	^{17}O	14.0	-14.7	-3.0	-4.2	-4.8	-2.3	-3.8	-4.7	-8.1	-4.1	-2.5	-3.2	-4.1	-4.5	-3.8	-4.4	-4.1	-9.6	-5.6
BeF	^{9}Be	294.0	-286.2	-279.1	-282.6	-285.0	-281.1	-286.6	-286.2	-290.6	-276.0	-280.3	-284.3	-282.4	-280.1	-285.0	-282.4	-285.4	-305.1	-286.3
	^{19}F	229.0	218.3	204.3	200.6	199.5	198.0	198.5	201.7	194.8	210.4	199.6	196.8	201.6	205.4	196.4	203.8	196.7	258.5	208.5
BeH	^{9}Be	199.3	-193.6	-190.6	-199.1	-203.0	-191.1	-199.4	-199.0	-208.1	-189.8	-188.9	-195.5	-193.8	-189.4	-196.7	-191.2	-197.2	-206.0	-194.7
	^{1}H	193.9	197.9	185.1	169.9	163.4	187.9	178.3	178.9	163.6	186.1	196.5	182.9	184.5	195.5	181.6	194.9	180.8	208.0	190.4
CH	^{1}H	57.7	-56.9	-96.6	-91.2	-85.7	-55.3	-58.2	-57.6	-61.4	-96.1	-56.5	-57.8	-57.3	-54.6	-57.8	-54.8	-57.6	-47.6	-59.6
	^{13}C	47.1	42.3	26.6	34.6	36.4	68.6	57.1	58.3	55.0	34.7	79.3	63.0	66.0	75.2	61.0	74.4	60.1	56.6	36.6
CO^+	^{13}C	1573.0	1538.0	1448.7	1492.1	1544.4	1438.8	1536.3	1513.3	1626.0	1479.7	1479.6	1514.0	1498.4	1478.9	1523.4	1487.9	1523.9	1603.5	1550.4
	^{17}O	18.5	19.2	32.4	32.7	31.6	30.4	33.1	32.2	28.9	32.3	32.0	33.3	32.1	30.2	32.4	30.2	32.1	30.7	33.6
Cl_2^-	^{35}Cl	109.0	125.3	121.6	121.2	121.3	122.3	122.1	123.5	120.2	118.7	120.0	121.2	123.0	118.0	120.2	118.7	120.2	95.4	121.8
OH	^{17}O	51.3	-51.7	-62.7	-60.1	-59.0	-92.7	-61.9	-62.4	-58.1	-66.8	-98.2	-66.9	-70.4	-89.3	-63.3	-89.0	-61.8	-45.5	-47.4
	^{1}H	71.5	-72.2	-64.8	-68.6	-71.4	-58.6	-69.9	-69.0	-74.5	-64.7	-61.6	-66.7	-66.0	-58.2	-66.4	-57.8	-66.3	-66.4	-75.0
SH	^{1}H	65.0	-51.4	-52.9	-47.2	-48.4	-40.1	-44.8	-44.4	-47.7	-86.7	-45.9	-46.9	-47.2	-45.0	-46.4	-44.8	-46.0	-40.3	-52.4
	^{33}S		36.9	122.2	42.1	38.4	52.8	42.9	45.3	35.3	58.0	50.6	42.4	45.0	47.1	41.3	48.0	41.5	17.3	26.4
BH_2	^{11}B	357.9	348.4	383.3	384.2	385.2	345.7	361.6	360.4	376.7	383.4	346.5	351.9	351.1	347.3	353.1	349.3	354.0	366.2	348.5
	^{1}H	38.0	35.5	14.7	14.0	15.3	40.1	34.7	35.2	28.5	14.7	37.4	34.3	34.3	37.7	33.9	37.2	33.9	43.4	32.5
BeOH	^{9}Be	264.0	-259.3	-254.3	-256.7	-259.7	-256.2	-261.0	-260.7	-264.4	-251.1	-254.9	-258.6	-256.7	-254.6	-259.4	-256.8	-259.8	-277.1	-259.5
	^{17}O		-46.1	-43.0	-42.2	-41.6	-42.7	-41.8	-42.0	-39.4	-42.8	-42.2	-41.9	-42.0	-43.0	-42.2	-42.8	-42.2	-54.6	-44.1
	^{1}H	< 5.0	2.4	2.3	2.2	2.2	2.7	2.4	2.4	2.0	2.0	2.5	2.5	2.4	2.4	2.4	2.4	2.4	3.7	2.2
CH_2	^{13}C		236.6	250.5	252.2	254.3	239.6	247.1	246.6	256.0	258.3	246.7	242.1	244.7	244.1	241.8	244.2	241.8	241.1	235.3
	^{1}H	20.2	-20.1	-29.2	-30.0	-29.7	-14.0	-19.5	-19.5	-25.6	-32.9	-17.1	-17.7	-19.0	-15.6	-17.0	-15.6	-16.9	-10.1	-23.2
CH_2^-	^{13}C	58.9	63.1	56.6	59.2	64.3	65.1	69.6	69.0	75.9	65.0	71.9	68.6	70.8	69.7	68.8	69.4	68.6	85.8	57.0
	^{1}H	44.8	-42.7	-48.1	-49.2	-50.0	-41.9	-46.0	-45.5	-50.2	-51.2	-45.2	-45.0	-45.1	-43.6	-45.0	-43.7	-44.9	-38.6	-47.1
C_2H	^{13}C	213.0	230.5	227.9	228.1	229.1	230.3	225.3	225.7	217.3	222.7	225.1	227.6	223.6	228.6	228.4	228.8	229.0	227.8	221.7
	^{13}C	1014.5	1025.0	995.3	1020.8	1043.1	1001.6	1043.2	1032.9	1091.2	1007.0	1024.9	1035.6	1028.0	1027.5	1037.0	1031.9	1037.0	1080.2	1038.7
	^{1}H	50.4	48.3	50.8	53.7	56.0	53.3	56.4	56.8	61.4	52.9	53.7	52.3	54.7	53.0	51.2	52.3	51.2	58.6	55.3
HCO	^{1}H	377.5	380.6	375.4	380.9	386.6	372.6	384.8	383.0	397.9	377.8	374.6	377.6	376.3	373.8	378.8	374.5	379.2	393.6	379.4
	^{1}H	354.0	359.4	348.1	350.2	352.2	353.9	358.6	357.3	362.2	351.6	357.3	356.5	355.1	355.1	356.9	353.8	357.0	379.1	361.6
	^{17}O	42.3	-44.5	-45.3	-45.6	-46.3	-47.2	-47.1	-48.1	-48.3	-48.8	-50.3	-47.9	-50.0	-48.0	-46.8	-46.9	-46.3	-35.8	-42.5
HCS	^{13}C		278.5	279.3	287.9	293.4	283.6	296.2	295.1	311.1	299.9	289.1	288.7	290.6	286.9	289.1	286.4	289.2	295.0	290.1
	^{1}H	127.5	129.1	120.0	118.4	118.6	122.8	123.5	123.1	120.1	111.2	122.9	125.1	123.9	123.3	124.9	122.7	124.8	135.3	118.3
	^{33}S		22.4	21.9	19.7	18.7	20.1	18.4	19.9	16.0	15.4	18.4	18.1	19.5	17.9	17.8	17.9	18.0	11.7	14.5
HOO	^{1}H	27.4	-23.9	-24.7	-25.0	-24.2	-22.4	-23.3	-23.5	-22.2	-24.6	-22.2	-23.4	-23.3	-21.7	-23.2	-21.6	-23.3	-21.6	-19.1
	^{17}O1		-59.6	-65.4	-64.6	-66.5	-67.4	-67.6	-67.3	-68.4	-70.2	-73.0	-68.5	-70.8	-65.2	-66.5	-63.4	-65.3	-45.8	-61.5
	^{17}O2		-34.8	-36.8	-36.3	-36.2	-40.3	-37.4	-38.5	-38.8	-41.9	-44.4	-37.8	-40.4	-41.5	-36.9	-40.4	-36.4	-27.7	-29.7
H_2O^+	^{17}O	83.2	-79.8	-86.2	-84.7	-84.1	-97.7	-88.7	-90.4	-88.6	-92.4	-107.7	-92.4	-97.3	-97.2	-89.2	-95.0	-87.9	-66.0	-77.0
	^{1}H	73.1	-75.2	-69.0	-71.8	-75.0	-67.3	-75.0	-73.8	-79.2	-69.5	-68.9	-70.8	-70.0	-65.9	-70.3	-65.9	-70.3	-70.8	-78.1
NH_2	^{1}H	67.2	-65.9	-65.8	-67.2	-68.9	-60.4	-66.5	-65.4	-71.2	-67.4	-63.0	-64.0	-63.5	-60.0	-63.6	-60.1	-63.5	-60.6	-69.3
	^{14}N	27.9	27.9	29.4	29.3	29.9	33.4	31.8	32.3	32.5	33.1	38.0	33.0	34.8	35.8	32.0	35.2	31.5	29.1	26.0
CH_3	^{13}C	75.7	74.0	72.6	75.7	79.5	79.7	84.9	84.3	92.3	84.5	90.8	83.7	86.0	86.4	83.0	85.5	82.4	83.3	72.1
	^{1}H	70.1	-69.4	-72.9	-75.5	-76.2	-65.7	-72.8	-72.8	-80.0	-77.0	-67.8	-69.2	-70.6	-65.1	-68.5	-65.0	-68.5	-64.8	-74.0
F_2CH	^{13}C	417.0	419.9	418.4	424.5	433.0	416.9	432.8	431.7	450.6	430.4	426.9	424.3	427.3	422.0	423.6	421.7	423.7	410.8	423.2
	^{19}F	236.0	240.3	249.3	248.9	249.9	265.6	257.4	262.0	265.3	265.6	278.2	257.9	267.7	268.2	251.9	264.0	250.2	219.6	232.0
	^{1}H	62.2	61.3	54.2	53.5	52.3	65.8	60.7	61.6	54.5	50.9	62.1	62.4	62.0	62.4	63.1	62.0	63.2	71.4	55.7
H_2CN	^{13}C	81.0	-74.9	-72.5	-75.5	-76.8	-73.4	-79.5	-78.9	-86.9	-76.3	-77.8	-77.5	-78.2	-75.3	-77.2	-75.4	-77.1	-68.1	-80.6
	^{1}H	233.2	216.1	215.8	213.3	211.5	216.6	213.8	213.5	210.7	222.2	224.3	221.4	221.8	223.8	221.5	223.2	221.5	232.1	219.9
	^{14}N	25.8	25.0	29.3	28.7	29.0	32.0	30.2	30.6	30.4	31.8	36.2	31.9	33.3	33.2	31.2	32.7	30.8	24.0	25.4
NH_3^+	^{14}N	54.9	45.5	41.3	42.9	45.0	46.3	48.1	48.1	51.0	46.5	51.7	48.1	49.8	48.7	47.1	47.8	46.7	42.0	44.8
	^{1}H	76.8	-80.7	-76.2	-78.6	-82.0	-75.1	-82.8	-81.1	-88.3	-78.6	-76.0	-78.5	-77.9	-73.1	-77.3	-73.1	-77.5	-75.9	-84.6
PH_3^+	^{31}P	1176.0	1181.2	1121.6	1146.9	1175.6	1163.3	1205.6	1203.1	1256.6	1147.4	1160.3	1160.9	1168.9	1146.6	1154.2	1145.2	1156.1	1077.6	1167.2
	^{1}H	< 5.9	3.0	2.4	1.6	1.1	13.0	7.6	7.8	3.2	-1.4	8.4	6.3	6.1	9.1	5.6	9.3	5.7	15.0	1.7
CH_2CH	^{13}C	24.0	-12.7	-11.2	-13.1	-14.0	-13.9	-18.0	-17.9	-24.5	-15.6	-18.8	-16.9	-18.7	-17.2	-16.5	-17.2	-16.3	-11.6	-19.7
	^{13}C	301.5	311.0	314.5	320.9	326.5	315.4	328.1	327.7	344.6	322.0	322.9	320.5	323.0	319.3	320.4	319.8	320.4	322.9	317.3
	^{1}H	192.0	163.0	165.1	163.3	161.7	165.7	164.1	163.5	162.1	170.2	171.9	169.0	169.5	171.6	168.5	171.0	168.4	179.0	166.1
	^{1}H	95.8	97.0	98.4	97.6	96.4	96.7	96.7	96.0	96.4	102.9	101.8	100.7	101.1	102.5	101.0	102.3	100.9	111.0	101.7
	^{1}H	37.3	43.8	37.7	35.8	35.5	47.0	41.9	41.1	34.2	33.9	44.7	46.1	43.1	45.7	46.8	45.6	46.8	55.8	37.3
H_2CCN	^{13}CCH2		67.2	68.5	71.6	75.7	72.0	79.2	78.6	88.7	76.3	82.4	77.4	80.0	78.5	77.2	77.8	76.8	71.3	72.6
	^{13}CCN		-59.7	-63.3	-65.0	-68.3	-62.4	-71.5	-69.8	-81.9	-64.0	-62.1	-64.9	-65.1	-60.6	-64.7	-60.7	-64.9	-60.1	-73.0
	^{1}H	58.8	-59.8	-65.6	-66.5	-68.4	-57.7	-65.7	-63.9	-73.3	-69.4	-59.8	-61.1	-60.8	-58.1	-60.7	-57.9	-60.7	-58.4	-67.3
	^{14}N	9.8	7.8	10.6	10.7	10.8	11.7	11.5	11.4	12.1	11.6	13.7	11.8	12.4	12.7	11.5	12.5	11.2	9.8	9.9
H_2CCO^+	^{13}CCH2		63.8	62.5	65.0	68.2	66.9	71.6	71.4	78.1	70.9	76.7	72.3	74.4	72.4	72.1	71.9	71.7	69.0	65.9
	^{13}CCO		-56.9	-56.8	-58.1	-61.2	-57.8	-64.0	-63.1	-71.7	-54.7	-54.3	-56.9	-57.2	-53.7	-56.9	-53.8	-57.4	-52.6	-67.6
	^{1}H	58.0	-60.2	-63.7	-64.4	-66.1	-57.4	-63.7	-62.7	-70.5	-66.9	-59.2	-59.6	-59.7	-57.2	-59.2	-57.1	-59.2	-58.6	-66.1
	^{17}O		-15.3	-20.5	-21.2	-21.5	-22.3	-22.4	-22.7	-23.6	-22.7	-24.6	-22.5	-23.9	-22.4	-21.8	-21.8	-21.4	-15.2	-19.5
MAE to CCSD(T) [MHz]				10.34	7.60	7.45	8.90	6.60	6.59	14.45	11.27	9.66	6.23	7.25	8.60	5.79	8.11	5.56	13.53	5.25
MAPE to CCSD(T) [%]				15.10	12.05	12.57	17.75	13.21	13.09	16.29	19.08	18.79	12.50	13.76	16.69	11.36	16.25	11.02	20.35	10.65
MAE to exp. [MHz]				13.18	11.12	10.92	12.80	9.55	9.49	15.88	13.04	12.40	9.14	9.81	11.82	8.66	11.31	8.49	14.58	7.63
MAPE to exp. [%]				14.41	13.62	13.78	14.70	11.01	10.73	13.31	16.21	15.55	11.67	12.10	14.27	11.08	13.94	10.76	14.16	9.53

Table S5: CBS extrapolated isotropic HFCCs for small main-group compounds.

Radical	Nuclei	Experiment	CBS CCSD(T)	RPA@TPSS	RPA@TPSSh	RPA@TPSS0	RPA@PBE	RPA@PBE0	RPA@B3LYP	RPA@ωB97X-V	σ(W1)@TPSS	σ(W1)@PBE	σ(W1)@PBE0	σ(W1)@B3LYP	σ(S1)@PBE	σ(S1)@PBE0	σ(S2)@PBE	σ(S2)@PBE0	B3LYP	DLPNO-CCSD
BO	^{11}B	1024.9	1031.1	993.5	1022.1	1044.2	987.3	1036.8	1028.6	1092.6	994.2	992.1	1016.6	1009.0	999.1	1022.3	1006.8	1025.0	1089.6	1048.6
	^{17}O	14.0	-14.7	-2.6	-4.4	-4.7	-2.3	-3.9	-4.7	-8.3	-3.7	-2.5	-3.3	-4.2	-4.6	-3.9	-4.6	-4.2	-9.6	-6.3
BeF	^{9}Be	294.0	-286.2	-280.1	-282.7	-284.8	-280.4	-286.3	-285.8	-290.5	-276.2	-278.9	-283.2	-280.9	-278.2	-283.7	-280.3	-284.2	-304.7	-286.4
	^{19}F	229.0	218.3	201.6	197.1	197.3	196.0	196.4	199.6	192.9	207.4	196.3	193.6	198.7	202.2	193.4	199.7	194.0	257.2	209.7
BeH	^{9}Be	199.3	-193.6	-188.2	-199.2	-202.9	-190.3	-198.9	-198.5	-208.0	-187.9	-187.4	-194.4	-192.5	-187.3	-195.8	-189.4	-196.5	-205.8	-194.8
	^{1}H	193.9	197.9	186.7	165.9	160.1	183.8	175.1	176.3	160.7	186.5	191.6	178.6	180.7	190.7	176.3	188.3	175.7	207.1	188.9
CH	^{1}H	57.7	-56.9	-95.9	-90.7	-85.5	-54.9	-58.0	-57.2	-61.3	-94.8	-56.2	-57.6	-56.8	-54.5	-57.6	-54.7	-57.5	-47.5	-59.5
	^{13}C	47.1	42.3	22.2	34.8	35.8	67.8	57.0	58.1	54.8	30.2	78.1	62.9	65.4	74.4	60.6	74.0	59.6	56.9	37.0
CO^{+}	^{13}C	1573.0	1538.0	1451.5	1492.4	1550.1	1438.6	1540.6	1515.7	1628.8	1473.0	1473.3	1516.6	1496.9	1472.9	1526.2	1485.5	1526.3	1603.6	1553.2
	^{17}O	18.5	19.2	32.3	33.3	30.5	31.2	32.4	32.3	28.8	32.6	33.0	32.5	32.3	31.4	31.6	31.2	31.2	30.7	32.8
Cl_2^{-}	^{35}Cl	109.0	125.3	121.0	120.7	120.8	121.8	121.5	123.1	119.9	118.6	119.8	120.8	122.8	117.6	119.5	117.9	119.6	94.8	120.1
OH	^{17}O	51.3	-51.7	-62.5	-60.1	-59.5	-105.0	-62.3	-61.3	-57.9	-67.1	-108.8	-67.7	-69.9	-101.5	-64.1	-102.1	-62.5	-45.6	-48.0
	^{1}H	71.5	-72.2	-64.5	-68.4	-71.2	-57.3	-69.8	-69.0	-74.5	-64.2	-61.5	-66.5	-65.9	-57.8	-66.2	-57.2	-66.2	-66.4	-74.9
SH	^{1}H	65.0	-51.4	-57.1	-47.0	-48.3	-40.2	-44.5	-44.2	-47.6	-107.6	-46.5	-46.6	-47.1	-46.4	-46.0	-46.1	-45.7	-40.3	-52.1
	^{33}S		36.9	166.9	42.0	38.6	54.1	43.1	45.7	35.3	68.1	53.4	42.7	45.6	49.4	41.3	50.3	41.6	16.9	26.8
BH_2	^{11}B	357.9	348.4	382.1	382.9	384.2	344.8	361.0	359.8	376.2	379.9	343.8	349.5	348.5	345.4	351.0	347.2	352.2	366.4	348.7
	^{1}H	38.0	35.5	15.8	14.0	15.0	39.9	34.3	34.8	28.1	16.8	37.4	33.9	34.0	37.9	33.4	37.2	33.4	43.4	32.5
BeOH	^{9}Be	264.0	-259.3	-254.4	-256.3	-259.4	-255.5	-260.7	-260.2	-264.2	-250.4	-253.5	-257.6	-255.2	-253.0	-258.1	-254.9	-258.6	-276.7	-259.7
	^{17}O		-46.1	-42.1	-41.4	-41.0	-42.1	-41.4	-41.5	-38.9	-41.9	-41.3	-41.2	-41.2	-42.1	-41.4	-41.6	-41.5	-54.4	-44.1
	^{1}H	< 5.0	2.4	2.1	2.1	2.2	2.6	2.4	2.3	2.0	1.9	2.4	2.4	2.3	2.2	2.3	2.2	2.3	3.7	2.2
CH_2	^{13}C		236.6	249.4	251.3	253.4	238.7	246.3	246.1	255.4	256.5	245.1	240.8	243.8	243.2	240.7	243.2	240.7	241.2	235.1
	^{1}H	20.2	-20.1	-29.5	-30.7	-30.0	-14.0	-19.5	-20.0	-25.7	-32.7	-17.1	-17.6	-19.4	-15.7	-17.0	-15.9	-16.9	-10.1	-23.0
CH_2^{-}	^{13}C	58.9	63.1	59.5	61.1	66.5	66.7	71.4	70.8	77.4	67.5	72.9	70.0	72.3	71.5	70.9	71.4	70.8	88.0	59.1
	^{1}H	44.8	-42.7	-47.7	-48.9	-49.6	-41.5	-45.3	-44.9	-49.7	-50.9	-44.7	-44.2	-44.5	-43.4	-44.3	-43.5	-44.2	-38.1	-46.6
C_2H	^{13}C	213.0	230.5	227.0	226.4	229.1	232.1	224.8	225.6	216.9	222.7	227.4	226.9	223.4	231.1	227.8	231.0	228.5	227.7	222.2
	^{13}C	1014.5	1025.0	996.0	1021.4	1042.4	1012.0	1044.1	1032.4	1092.0	1003.4	1034.0	1035.6	1025.4	1038.4	1036.6	1044.2	1036.2	1080.3	1038.8
	^{1}H	50.4	48.3	50.5	53.0	55.8	54.8	56.2	56.6	61.2	53.4	55.9	51.9	54.6	55.3	50.7	54.2	50.8	58.6	55.1
HCO	^{1}H	377.5	380.6	374.5	380.1	385.8	372.0	384.1	382.4	397.6	375.6	372.7	376.1	374.4	373.0	377.3	373.6	377.8	393.4	379.4
	^{1}H	354.0	359.4	347.4	349.6	351.6	352.9	358.0	356.7	361.5	352.3	356.8	355.7	354.5	354.0	355.9	351.9	356.1	378.9	361.2
	^{17}O	42.3	-44.5	-45.2	-45.5	-46.3	-47.1	-47.1	-48.0	-48.2	-48.9	-50.3	-48.0	-50.0	-48.1	-46.9	-46.9	-46.5	-35.9	-42.9
HCS	^{13}C		278.5	276.8	287.2	293.3	287.8	296.2	295.2	311.1	305.9	292.2	288.5	290.5	278.5	289.1	273.6	289.2	294.7	290.8
	^{1}H	127.5	129.1	120.2	118.0	117.9	116.7	122.8	122.5	119.6	106.9	120.6	124.2	123.3	133.8	123.8	136.5	123.8	135.2	118.1
	^{33}S		22.4	21.3	19.4	18.4	14.0	18.1	19.8	15.8	11.8	21.2	17.9	19.4	24.7	17.4	22.8	17.5	11.5	14.6
HOO	^{1}H	27.4	-23.9	-24.6	-25.0	-24.1	-22.5	-23.2	-23.4	-22.2	-24.5	-22.4	-23.4	-23.2	-22.0	-23.2	-21.9	-23.3	-21.6	-19.1
	^{17}O1		-59.6	-65.6	-65.0	-66.7	-68.0	-67.9	-66.6	-68.3	-70.6	-74.1	-69.0	-70.2	-65.8	-67.1	-64.2	-65.9	-45.9	-62.2
	^{17}O2		-34.8	-36.8	-36.3	-36.2	-40.4	-37.4	-38.4	-38.8	-42.0	-44.7	-37.9	-40.4	-41.5	-37.1	-40.6	-36.6	-27.7	-29.7
H_2O^{+}	^{17}O	83.2	-79.8	-85.8	-84.7	-83.8	-97.9	-88.5	-90.4	-88.5	-92.1	-108.2	-92.5	-97.4	-97.0	-89.8	-95.1	-88.5	-66.0	-77.6
	^{1}H	73.1	-75.2	-68.7	-71.4	-74.8	-67.1	-74.9	-73.6	-79.0	-69.3	-68.8	-70.7	-69.9	-65.8	-70.2	-65.8	-70.2	-70.7	-78.0
NH_2	^{1}H	67.2	-65.9	-65.6	-67.0	-68.7	-60.4	-66.3	-65.2	-71.0	-67.0	-62.9	-63.8	-63.2	-60.0	-63.4	-60.1	-63.4	-60.6	-69.2
	^{14}N	27.9	27.9	29.3	29.3	29.8	33.2	31.8	32.3	32.5	32.9	37.7	33.0	34.7	36.0	32.0	35.3	31.6	29.1	25.9
CH_3	^{13}C	75.7	74.0	72.0	75.3	78.9	79.4	84.5	83.9	92.0	83.7	90.0	83.3	85.2	86.0	82.5	85.3	81.9	83.5	71.1
	^{1}H	70.1	-69.4	-72.9	-76.2	-76.1	-65.3	-72.5	-73.3	-80.0	-77.1	-67.5	-68.8	-71.2	-64.7	-68.2	-64.6	-68.2	-64.8	-73.8
F_2CH	^{13}C	417.0	419.9	416.5	423.2	432.2	415.5	432.3	431.0	450.5	427.9	424.3	422.4	425.1	419.2	422.0	418.7	422.3	410.8	423.2
	^{19}F	236.0	240.3	249.0	248.4	249.4	265.9	256.9	261.6	264.9	266.2	279.6	258.1	268.1	270.1	251.9	265.4	250.3	219.5	231.0
	^{1}H	62.2	61.3	54.4	53.5	52.2	66.1	60.7	61.7	54.3	51.9	62.7	62.4	62.3	62.9	62.9	62.2	63.1	71.4	55.7
H_2CN	^{13}C	81.0	-74.9	-72.3	-75.6	-76.6	-72.7	-79.2	-78.9	-86.8	-75.9	-77.2	-77.1	-78.0	-74.7	-76.8	-74.7	-76.8	-68.0	-80.0
	^{1}H	233.2	216.1	216.2	213.4	211.8	217.7	214.2	213.7	210.8	223.0	225.6	222.3	222.3	225.0	222.4	224.5	222.3	232.0	219.9
	^{14}N	25.8	25.0	29.3	28.5	29.0	32.5	30.3	30.5	30.3	31.6	36.5	31.9	33.2	33.5	31.3	33.1	30.9	24.0	25.3
NH_3^{+}	^{14}N	54.9	45.5	40.9	42.7	44.9	46.3	47.9	48.1	50.9	46.4	51.6	47.9	49.7	48.9	46.9	48.1	46.5	42.1	44.6
	^{1}H	76.8	-80.7	-76.0	-78.0	-81.6	-74.9	-82.9	-80.9	-88.1	-78.4	-75.7	-78.6	-77.7	-72.9	-77.5	-72.8	-77.6	-75.8	-84.5
PH_3^{+}	^{31}P	1176.0	1181.2	1116.6	1145.2	1172.0	1154.6	1201.4	1200.2	1254.8	1143.1	1151.2	1155.8	1165.8	1138.6	1149.7	1136.0	1152.2	1075.9	1165.5
	^{1}H	< 5.9	3.0	2.1	1.2	0.7	13.4	7.5	7.5	2.9	-1.7	8.6	6.1	5.7	9.3	5.3	9.4	5.5	15.0	1.9
CH_2CH	^{13}C	24.0	-12.7	-11.2	-13.6	-13.9	-14.5	-17.9	-18.4	-24.7	-15.5	-19.2	-16.9	-19.2	-17.7	-16.5	-17.9	-16.4	-11.6	-19.4
	^{13}C	301.5	311.0	313.5	320.6	325.5	315.7	327.3	327.9	344.2	320.2	322.2	319.0	322.3	319.2	319.1	319.8	319.2	323.0	316.4
	^{1}H	192.0	163.0	165.4	163.3	161.9	165.1	164.3	163.2	162.1	171.1	171.9	169.4	169.6	171.5	168.9	170.8	168.7	178.9	166.1
	^{1}H	95.8	97.0	98.9	98.0	96.7	96.7	97.0	96.2	96.6	103.2	102.0	101.2	101.5	102.8	101.6	102.8	101.5	110.9	101.8
	^{1}H	37.3	43.8	37.5	35.0	35.5	45.9	42.0	40.3	34.1	34.2	43.7	46.0	42.1	44.5	46.6	44.2	46.6	55.8	37.4
H_2CCN	^{13}CCH2		67.2	68.2	71.1	75.3	71.8	79.0	78.4	88.5	74.6	82.1	77.0	79.6	78.1	77.0	77.5	76.7	71.5	72.1
	^{13}CCN		-59.7	-64.2	-64.7	-67.9	-62.4	-71.7	-69.6	-81.8	-64.8	-61.8	-65.0	-64.7	-60.4	-64.9	-60.7	-65.1	-60.1	-72.6
	^{1}H	58.8	-59.8	-66.7	-66.5	-68.3	-57.5	-65.9	-63.8	-73.2	-70.4	-59.7	-61.4	-60.4	-58.0	-60.9	-57.8	-61.0	-58.4	-67.2
	^{14}N	9.8	7.8	10.4	10.7	10.7	11.7	11.4	11.4	12.1	11.2	13.7	11.8	12.4	12.7	11.4	12.6	11.2	9.8	9.8
H_2CCO^{+}	^{13}CCH2		63.8	62.0	64.5	67.8	66.5	71.5	71.2	77.8	70.3	76.2	72.0	74.0	71.2	72.0	70.8	71.7	69.1	65.6
	^{13}CCO		-56.9	-57.2	-58.0	-60.8	-58.0	-63.7	-63.2	-71.6	-54.9	-54.6	-56.6	-57.2	-54.0	-56.6	-54.2	-57.1	-52.6	-67.1
	^{1}H	58.0	-60.2	-64.1	-64.4	-65.9	-57.3	-63.4	-62.7	-70.4	-67.1	-59.3	-59.2	-59.6	-57.4	-58.8	-57.2	-58.9	-58.5	-66.0
	^{17}O		-15.3	-20.3	-21.1	-21.5	-22.2	-22.4	-22.6	-23.5	-22.7	-24.5	-22.6	-23.9	-22.3	-21.9	-21.7	-21.5	-15.2	-19.4
MAE to CCSD(T) [MHz]				11.30	7.70	7.62	9.66	6.61	6.53	14.54	12.33	10.60	6.45	7.48	9.38	6.05	9.03	5.79	13.57	5.25
MAPE to CCSD(T) [%]				17.54	12.47	12.80	19.20	13.17	13.05	16.29	20.77	19.45	12.53	13.80	17.47	11.48	17.02	11.14	20.47	10.21
MAE to exp. [MHz]				13.20	11.23	10.94	13.49	9.47	9.44	15.99	13.98	13.46	9.47	10.14	12.95	8.96	12.58	8.70	14.63	7.56
MAPE to exp. [%]				14.39	13.78	13.80	15.52	11.01	10.66	13.31	17.07	16.22	11.83	12.14	15.06	11.22	14.80	10.92	14.27	9.26

Table S6: Isotropic HFCCs for small main-group compounds with pcJ-4 and different auxiliary basis sets.

			RPA@B3LYP				$\sigma(S2)$@PBE0			
Radical	Nuclei	CBS CCSD(T)	cc-pwcVDZ-RI	cc-pwcVTZ-RI	cc-pwcVQZ-RI	cc-pwcV5Z-RI	cc-pwcVDZ-RI	cc-pwcVTZ-RI	cc-pwcVQZ-RI	cc-pwcV5Z-RI
BO	^{11}B	1031.1	1022.4	1024.9	1027.1	1027.5	1026.6	1028.2	1027.3	1026.7
	^{17}O	-14.7	-4.5	-4.7	-4.7	-4.7	-3.9	-4.0	-4.1	-4.1
CH	^{1}H	-56.9	-57.6	-57.6	-57.6	-57.6	-57.7	-57.6	-57.7	-57.6
	^{13}C	42.3	58.8	58.3	58.2	58.3	60.5	59.9	60.1	60.1
CO^+	^{13}C	1538.0	1507.0	1510.3	1512.7	1513.3	1517.5	1520.5	1524.2	1523.9
	^{17}O	19.2	32.5	32.3	32.2	32.2	32.7	32.4	32.1	32.1
Cl_2^-	^{35}Cl	125.3	123.7	123.7	123.5	123.5	120.1	120.4	120.3	120.2
OH	^{17}O	-51.7	-62.4	-62.4	-62.4	-62.4	-61.2	-61.4	-61.8	-61.8
	^{1}H	-72.2	-69.2	-69.1	-69.0	-69.0	-66.5	-66.4	-66.4	-66.3
SH	^{1}H	-51.4	-44.4	-44.4	-44.4	-44.4	-46.3	-46.1	-46.1	-46.0
	^{33}S	36.9	45.2	45.3	45.2	45.3	41.2	41.6	41.6	41.5
BH_2	^{11}B	348.4	358.8	359.5	360.2	360.4	356.9	355.9	354.8	354.0
	^{1}H	35.5	35.6	35.3	35.1	35.2	35.1	34.2	34.1	33.9
CH_2	^{13}C	236.6	246.5	246.3	246.5	246.6	243.4	242.2	242.2	241.8
	^{1}H	-20.1	-19.7	-19.6	-19.6	-19.5	-16.7	-16.7	-16.9	-16.9
CH_2^-	^{13}C	63.1	67.5	66.9	67.0	69.0	65.7	65.4	66.4	68.6
	^{1}H	-42.7	-45.9	-45.8	-45.7	-45.5	-45.5	-45.2	-45.1	-44.9
C_2H	^{13}C	230.5	226.3	226.0	225.7	225.7	231.1	229.8	229.2	229.0
	^{13}C	1025.0	1027.3	1029.9	1032.2	1032.9	1032.8	1036.4	1037.8	1037.0
	^{1}H	48.3	57.4	57.0	56.9	56.8	52.6	51.8	51.3	51.2
HCO	^{1}H	380.6	381.8	382.3	382.8	383.0	381.7	380.3	380.0	379.2
	^{1}H	359.4	358.2	357.5	357.2	357.3	359.5	357.9	357.3	357.0
	^{17}O	-44.5	-48.3	-48.2	-48.1	-48.1	-46.4	-46.3	-46.4	-46.3
HCS	^{13}C	278.5	294.1	294.7	295.0	295.1	288.8	289.5	289.7	289.2
	^{1}H	129.1	123.8	123.4	123.1	123.1	126.3	125.3	124.9	124.8
	^{33}S	22.4	20.2	20.0	19.9	19.9	18.6	18.2	18.0	18.0
HOO	^{1}H	-23.9	-23.7	-23.5	-23.5	-23.5	-23.4	-23.3	-23.3	-23.3
	^{17}O1	-59.6	-67.5	-67.4	-67.3	-67.3	-65.0	-65.0	-65.3	-65.3
	^{17}O2	-34.8	-38.7	-38.5	-38.4	-38.5	-36.5	-36.4	-36.5	-36.4
H_2O^+	^{17}O	-79.8	-90.7	-90.5	-90.4	-90.4	-87.7	-87.7	-88.0	-87.9
	^{1}H	-75.2	-74.0	-73.8	-73.8	-73.8	-70.5	-70.4	-70.4	-70.3
NH_2	^{1}H	-65.9	-65.5	-65.5	-65.4	-65.4	-63.7	-63.6	-63.6	-63.5
	^{14}N	27.9	32.4	32.3	32.3	32.3	31.5	31.4	31.6	31.5
CH_3	^{13}C	74.0	85.0	84.4	84.3	84.3	83.4	82.6	82.5	82.4
	^{1}H	-69.4	-72.9	-72.8	-72.8	-72.8	-68.9	-68.6	-68.6	-68.5
F_2CH	^{13}C	419.9	430.3	430.9	431.5	431.7	425.4	425.1	424.4	423.7
	^{19}F	240.3	262.7	262.2	262.0	262.0	250.7	250.3	250.4	250.2
	^{1}H	61.3	61.9	61.7	61.6	61.6	63.7	63.3	63.2	63.2
H_2CN	^{13}C	-74.9	-79.3	-79.0	-78.9	-78.9	-77.5	-77.2	-77.1	-77.1
	^{1}H	216.1	214.5	213.9	213.6	213.5	222.0	221.5	221.4	221.5
	^{14}N	25.0	30.7	30.6	30.5	30.6	30.7	30.6	30.8	30.8
NH_3^+	^{14}N	45.5	48.4	48.2	48.1	48.1	46.9	46.7	46.7	46.7
	^{1}H	-80.7	-81.2	-81.1	-81.1	-81.1	-77.7	-77.6	-77.6	-77.5
PH_3^+	^{31}P	1181.2	1200.6	1201.5	1202.1	1203.1	1163.1	1159.8	1158.6	1156.1
	^{1}H	3.0	7.5	7.7	7.8	7.8	6.1	5.8	5.8	5.7
CH_2CH	^{13}C	-12.7	-18.3	-18.0	-17.9	-17.9	-16.3	-16.2	-16.3	-16.3
	^{13}C	311.0	326.9	327.1	327.5	327.7	321.6	320.9	320.9	320.4
	^{1}H	163.0	164.5	163.8	163.6	163.5	169.3	168.6	168.3	168.4
	^{1}H	97.0	96.8	96.3	96.1	96.0	100.7	100.7	100.8	100.9
	^{1}H	43.8	41.1	41.1	41.1	41.1	46.6	46.7	46.7	46.8
H_2CCN	^{13}CCH2	67.2	79.2	78.7	78.6	78.6	77.5	76.9	76.8	76.8
	^{13}CCN	-59.7	-69.9	-69.8	-69.8	-69.8	-65.2	-64.9	-65.0	-64.9
	^{1}H	-59.8	-64.0	-63.9	-63.9	-63.9	-61.0	-60.9	-60.8	-60.7
	^{14}N	7.8	11.5	11.4	11.4	11.4	11.2	11.2	11.3	11.2
H_2CCO^+	^{13}CCH2	63.8	72.1	71.6	71.4	71.4	72.6	71.9	71.8	71.7
	^{13}CCO	-56.9	-63.4	-63.2	-63.1	-63.1	-57.6	-57.4	-57.4	-57.4
	^{1}H	-60.2	-62.9	-62.7	-62.7	-62.7	-59.5	-59.3	-59.3	-59.2
	^{17}O	-15.3	-22.8	-22.7	-22.7	-22.7	-21.4	-21.4	-21.4	-21.4
MAE to CCSD(T) [MHz]			6.57	6.51	6.52	6.59	5.40	5.33	5.36	5.41
MAPE to CCSD(T) [%]			14.15	14.06	14.06	14.14	12.04	11.74	11.80	11.82

Table S7: Isotropic HFCCs for large main-group compounds using the pcJ basis sets. The aug-cc-pVTZ-J data is taken from *J. Chem. Phys.* **2022**, *156*, 094107.

			aug-cc-pVTZ-J	pcJ-2					pcJ-3					pcJ-[2,3] extrapolated				
Radical	Nuclei	Experiment	CCSD	B3LYP	RPA@B3LYP	RPA@PBE0	$\sigma(S2)$@PBE0	DLPNO-CCSD	B3LYP	RPA@B3LYP	RPA@PBE0	$\sigma(S2)$@PBE0	DLPNO-CCSD	B3LYP	RPA@B3LYP	RPA@PBE0	$\sigma(S2)$@PBE0	DLPNO-CCSD
Aniline+	^{13}C1, ^{13}C5		-25.2	-18.9	-22.2	-23.4	-20.0	-27.6	-19.0	-22.5	-23.7	-20.5	-27.6	-19.0	-22.6	-23.9	-20.9	-27.6
	^{13}C2, ^{13}C4		12.5	12.2	12.4	12.9	11.1	15.8	12.1	12.7	13.2	11.6	15.8	12.1	12.8	13.4	11.8	15.7
	^{13}C3		-19.4	-12.9	-15.6	-16.8	-12.7	-20.0	-13.0	-15.7	-17.0	-13.1	-19.5	-13.1	-15.8	-17.0	-13.3	-19.2
	^{13}C6		35.2	32.5	34.9	35.6	33.6	37.0	32.4	35.4	36.1	34.2	36.7	32.3	35.6	36.4	34.6	36.5
	^{1}H7, ^{1}H10	4.3	4.0	3.9	3.1	3.6	1.6	5.6	3.9	3.2	3.7	1.8	5.3	3.9	3.2	3.7	1.9	5.2
	^{1}H8,^{1}H9	16.3	-15.8	-14.1	-13.9	-14.4	-12.4	-17.5	-14.2	-14.0	-14.6	-12.6	-17.0	-14.2	-14.1	-14.6	-12.7	-16.7
	^{1}H11	26.8	-33.8	-28.2	-29.5	-30.6	-27.6	-35.4	-28.4	-29.7	-31.0	-28.1	-34.8	-28.4	-29.9	-31.1	-28.3	-34.4
	^{1}H13, ^{1}H14	26.8	-28.7	-26.5	-28.0	-28.4	-25.5	-28.2	-26.5	-27.7	-28.2	-25.3	-27.5	-26.5	-27.6	-28.1	-25.1	-27.2
	^{14}N12	21.5	17.2	14.6	17.9	18.0	16.6	16.4	14.6	17.9	18.0	16.6	16.5	14.6	17.9	18.1	16.7	16.6
4-Nitroaniline+	^{13}C1, ^{13}C5		-28	-20.3	-25.0	-26.2	-22.5	-28.6	-20.3	-24.6	-25.4	-21.8	-29.0	-20.3	-24.3	-25.0	-21.4	-29.3
	^{13}C2, ^{13}C4		16.4	14.6	15.8	16.4	14.2	19.5	14.5	15.6	16.1	13.9	19.3	14.4	15.4	15.9	13.8	19.3
	^{13}C3		-24.2	-16.1	-20.6	-22.0	-17.4	-23.6	-16.1	-19.8	-20.7	-16.2	-23.0	-16.2	-19.4	-20.0	-15.5	-22.7
	^{13}C6		48.8	40.2	46.5	47.4	43.8	49.4	40.1	46.6	47.3	43.8	49.2	40.0	46.7	47.3	43.8	49.0
	^{1}H7, ^{1}H10	5.8	6.5	5.7	5.4	6.1	3.7	7.7	5.7	5.0	5.3	2.9	7.5	5.7	4.8	4.9	2.5	7.3
	^{1}H8, ^{1}H9	18.0	-17.9	-15.3	-16.1	-16.7	-14.3	-19.3	-15.3	-15.6	-15.9	-13.5	-18.8	-15.3	-15.4	-15.5	-13.1	-18.4
	^{1}H12, ^{1}H13	28.7	-29.2	-26.6	-28.8	-29.3	-26.1	-28.3	-26.6	-28.3	-28.5	-25.3	-27.6	-26.6	-28.0	-28.1	-24.9	-27.2
	^{14}N11	22.4	17.6	14.6	18.2	18.3	16.8	17.1	14.6	18.2	18.3	16.7	17.2	14.6	18.1	18.2	16.7	17.3
	^{14}N14	5.8	-7.6	-5.1	-6.5	-6.9	-6.0	-7.6	-5.2	-6.5	-6.8	-5.9	-7.8	-5.3	-6.5	-6.7	-5.9	-7.9
	^{17}O15, ^{17}O16		-2.1	-2.0	-2.4	-2.4	-2.3	-2.3	-2.0	-2.5	-2.5	-2.5	-2.1	-2.0	-2.6	-2.6	-2.7	-2.0
Benzyl	^{13}C1, ^{13}C2		22.9	20.5	23.2	23.6	20.8	23.6	20.4	22.9	23.7	21.1	23.4	20.4	22.8	23.8	21.2	23.4
	^{13}C3, ^{13}C5		-22.5	-18.5	-21.9	-22.2	-19.0	-23.6	-18.5	-21.5	-22.4	-19.3	-23.8	-18.5	-21.3	-22.5	-19.5	-23.9
	^{13}C4		23.8	21.8	24.0	24.3	21.8	23.5	21.8	24.0	24.5	22.1	23.2	21.7	24.0	24.7	22.3	23.0
	^{13}C11	40.5	-50.4	-37.8	-47.8	-48.7	-43.8	-50.4	-37.9	-46.9	-48.9	-44.3	-49.5	-38.0	-46.3	-48.9	-44.5	-48.9
	^{13}C12	68.5	63.6	61.0	66.7	67.9	65.5	62.0	60.7	67.1	68.0	65.6	62.4	60.5	67.3	68.1	65.6	62.7
	^{1}H6, ^{1}H7	14.4	-15.9	-14.7	-15.5	-15.5	-13.2	-16.7	-14.8	-14.9	-15.5	-13.3	-16.3	-14.8	-14.5	-15.4	-13.3	-16.1
	^{1}H8, ^{1}H10	5.0	6.1	6.1	6.2	6.3	4.3	7.1	6.1	5.8	6.3	4.3	6.9	6.1	5.6	6.3	4.4	6.8
	^{1}H9	17.3	-19.5	-17.3	-18.3	-18.2	-16.0	-19.6	-17.4	-18.0	-18.5	-16.4	-19.3	-17.5	-17.8	-18.7	-16.6	-19.2
	^{1}H13, ^{1}H14	45.7	-55.1	-48.0	-53.9	-54.6	-50.4	-57.0	-48.1	-52.4	-54.3	-50.3	-55.6	-48.1	-51.5	-54.2	-50.3	-54.8
Phenylaminyl	^{13}C1		-27.7	-21.1	-25.0	-26.4	-22.8	-27.7	-21.2	-25.4	-26.2	-22.8	-27.9	-21.2	-25.6	-26.1	-22.8	-28.1
	^{13}C2		25.6	21.7	24.2	25.4	22.4	26.6	21.7	24.6	25.2	22.4	26.6	21.7	24.8	25.1	22.3	26.5
	^{13}C3		-50.8	-38.0	-45.1	-47.6	-41.7	-50.5	-37.9	-45.5	-47.1	-41.6	-49.6	-37.9	-45.8	-46.8	-41.6	-49.1
	^{13}C4		24.1	20.9	23.4	24.5	21.7	25.3	20.7	23.7	24.3	21.6	25.2	20.7	23.9	24.2	21.5	25.1
	^{13}C5		-27.9	-21.5	-25.3	-26.7	-23.1	-27.9	-21.5	-25.6	-26.5	-23.0	-28.2	-21.5	-25.8	-26.3	-23.0	-28.3
	^{13}C6		30.4	26.3	29.4	30.3	27.7	28.5	26.2	29.8	30.3	27.8	28.2	26.2	30.1	30.3	28.0	28.0
	^{1}H7	5.6	7.9	6.9	6.6	7.4	5.1	8.3	6.9	6.8	7.1	4.8	8.0	7.0	6.9	6.9	4.7	7.9
	^{1}H8	17.3	-19.5	-17.0	-17.5	-18.4	-15.9	-19.6	-17.0	-17.6	-18.0	-15.6	-19.1	-17.0	-17.6	-17.7	-15.4	-18.9
	^{1}H9	17.3	-19.7	-17.4	-17.7	-18.6	-16.1	-19.9	-17.4	-17.8	-18.2	-15.8	-19.3	-17.3	-17.9	-18.0	-15.6	-19.0
	^{1}H10	5.6	8	7.1	6.8	7.6	5.3	8.4	7.1	7.0	7.3	5.0	8.1	7.1	7.1	7.1	4.9	8.0
	^{1}H11	23.0	-24.8	-20.8	-22.2	-23.0	-20.6	-24.0	-21.0	-22.6	-23.1	-20.8	-23.8	-21.1	-22.9	-23.1	-20.9	-23.6
	^{1}H13	36.3	-45.6	-39.5	-41.4	-42.9	-38.9	-47.7	-39.6	-41.3	-42.5	-38.6	-46.5	-39.6	-41.2	-42.2	-38.4	-45.7
	^{14}N12	22.3	24.5	20.9	24.8	24.9	23.4	23.9	21.1	25.3	25.5	24.2	24.3	21.1	25.6	25.8	24.7	24.5
1,3,2-benzodithiazolyl	^{13}C1, ^{13}C2		-7.1	-5.3	-6.4	-6.4	-6.6	-7.5	-5.1	-6.1	-6.4	-5.5	-7.4	-5.0	-5.9	-6.3	-4.8	-7.4
	^{13}C3, ^{13}C6		0.0	0.8	0.5	0.4	0.6	0.3	0.8	0.3	0.2	0.4	0.1	0.8	0.2	0.1	0.4	0.0
	^{13}C4, ^{13}C5		-0.5	-0.1	-0.3	-0.4	-0.2	0.1	-0.2	-0.3	-0.3	-0.2	0.0	-0.2	-0.3	-0.3	-0.3	0.0
	^{1}H7, ^{1}H10	-1.7	-1.3	-1.9	-1.6	-1.6	-1.6	-1.2	-1.8	-1.5	-1.4	-1.7	-1.2	-1.8	-1.4	-1.2	-1.7	-1.1
	^{1}H8, ^{1}H9	-1.7	-1.2	-1.4	-1.4	-1.3	-1.4	-1.2	-1.5	-1.4	-1.4	-1.4	-1.1	-1.5	-1.4	-1.4	-1.5	-1.1
	^{33}S11, ^{13}S12	10.9	9.9	7.3	13.3	13.2	12.1	9.9	6.3	11.4	11.5	9.5	7.0	5.6	10.3	10.6	8.0	5.3
	^{14}N13	30.8	36.3	27.8	34.6	34.7	31.9	36.2	27.2	34.6	34.0	32.6	35.8	26.9	34.5	33.7	33.0	35.6
Diethylaminyl	^{13}C1, ^{13}C9		-2.4	-2.0	-1.7	-1.6	-1.6	-2.0	-2.0	-1.7	-1.7	-1.7	-2.1	-2.0	-1.7	-1.8	-1.7	-2.1
	^{13}C2, ^{13}C8		-37.9	-29.5	-35.8	-36.4	-35.5	-38.1	-29.6	-35.6	-36.1	-35.3	-38.0	-29.7	-35.5	-35.8	-35.2	-38.0
	^{1}H3, ^{1}H12		-3.6	-3.1	-3.2	-3.3	-3.0	-3.7	-3.1	-3.2	-3.3	-3.0	-3.6	-3.1	-3.2	-3.3	-3.0	-3.6
	^{1}H4, ^{1}H5, ^{1}H13, ^{1}H14		-1.8	-1.5	-1.9	-1.9	-1.7	-1.9	-1.5	-1.8	-1.8	-1.7	-1.9	-1.5	-1.8	-1.8	-1.6	-1.9
	^{1}H6, ^{1}H7, ^{1}H10, ^{1}H11	103.4	98.4	111.3	96.2	96.7	99.5	99.2	111.8	97.1	97.8	101.3	99.8	112.1	97.7	98.4	102.3	100.2
	^{14}N15	40.0	40.3	34.1	41.2	40.7	39.9	38.2	34.3	42.0	41.6	40.9	38.3	34.4	42.5	42.0	41.4	38.4
Cyclo-hexyl	^{13}C1		-1.8	-0.6	-2.1	-2.0	-1.7	-1.8	-0.7	-2.0	-2.0	-1.6	-1.8	-0.7	-1.9	-2.0	-1.6	-1.7
	^{13}C2, ^{13}C6		21.9	23.5	21.7	21.9	22.2	22.1	23.6	21.8	21.9	22.3	22.4	23.6	21.8	21.9	22.3	22.5
	^{13}C3, ^{13}C5		-33.1	-24.1	-31.6	-31.6	-30.0	-33.5	-24.2	-31.3	-31.8	-30.5	-33.5	-24.2	-31.2	-31.9	-30.7	-33.5
	^{13}C4	115.7	119.9	115.2	124.7	125.1	123.5	116.9	114.9	125.3	125.6	123.7	117.5	114.7	125.7	125.9	123.8	117.8
	^{1}H7, ^{1}H13	14.9	15.5	17.3	15.6	15.6	15.5	15.9	17.3	15.5	15.7	15.8	15.9	17.3	15.5	15.8	15.9	15.9
	^{1}H8, ^{1}H15	2.0	-3.1	-2.5	-2.9	-2.9	-2.8	-3.1	-2.5	-2.9	-2.9	-2.8	-3.0	-2.5	-2.9	-2.9	-2.8	-3.0
	^{1}H9, ^{1}H16	2.0	-0.1	0.9	-0.1	0.0	0.2	-0.1	0.9	-0.1	0.0	0.2	-0.1	0.9	-0.1	-0.1	0.2	-0.1
	^{1}H10		-0.1	0.2	-0.1	-0.1	-0.1	0.0	0.1	-0.1	-0.1	-0.1	0.0	0.1	-0.1	-0.1	0.0	0.0
	^{1}H11		2.2	2.8	2.0	2.1	2.2	2.2	2.8	2.0	2.1	2.2	2.2	2.8	2.0	2.1	2.2	2.2
	^{1}H12	59.7	-63.7	-54.2	-62.5	-62.3	-58.2	-65.3	-54.3	-61.7	-62.7	-58.8	-64.4	-54.4	-61.3	-63.0	-59.1	-63.8
	^{1}H14, ^{1}H17	110.4	117.5	129.7	116.0	116.8	118.4	117.9	130.1	116.5	117.2	119.1	118.4	130.3	116.8	117.3	119.5	118.7
1-adamantyl	^{13}C1, ^{13}C7, ^{13}C21		-23.1	-16.8	-21.0	-20.9	-19.4	-22.8	-16.9	-20.5	-21.1	-19.6	-22.5	-16.9	-20.2	-21.1	-19.7	-22.4
	^{13}C4, ^{13}C13, ^{13}C24		38.8	38.3	39.1	39.1	39.1	38.6	38.5	39.0	39.0	38.9	38.7	38.5	39.0	39.0	38.8	38.8
	^{13}C6		223.4	211.1	224.0	222.5	220.1	219.5	211.1	224.3	224.8	221.6	221.0	211.1	224.5	226.2	222.5	222.0
	^{13}C10, ^{13}C15, ^{13}C18		-4.7	-3.8	-4.5	-4.4	-4.2	-4.2	-3.8	-4.5	-4.5	-4.2	-4.2	-3.9	-4.4	-4.5	-4.3	-4.2
	^{1}H2, ^{1}H3, ^{1}H8, ^{1}H9, ^{1}H22, ^{1}H23	18.4	17.5	19.6	17.1	17.1	17.1	17.7	19.6	17.1	17.3	17.4	17.7	19.7	17.1	17.4	17.5	17.8
	^{1}H5, ^{1}H14, ^{1}H25	13.1	10.9	13.1	11.0	11.4	11.9	10.9	13.1	11.2	11.3	11.7	10.9	13.1	11.3	11.2	11.6	11.0
	^{1}H11, ^{1}H17, ^{1}H20	8.6	6.7	8.9	6.4	6.7	7.2	6.3	8.9	6.5	6.6	7.2	6.3	9.0	6.6	6.6	7.2	6.3
	^{1}H12,^{1}H16, ^{1}H19	2.2	1.9	2.4	1.8	1.9	1.9	1.8	2.4	1.8	1.8	1.9	1.8	2.4	1.8	1.8	1.9	1.8
MAE to CCSD [MHz]				3.70	1.30	0.95	2.48	0.78	3.73	1.35	1.04	2.47	0.76	3.75	1.39	1.11	2.52	0.78
MAE to exp. [MHz]			2.83	2.57	2.36	2.48	2.13	3.19	2.64	2.24	2.44	2.24	3.02	2.68	2.21	2.45	2.34	2.92

Cartesian coordinates for small main-group compounds

Cartesian coordinates are given in Å and the xyz format with charge and multiplicity.

BO

```
2
0 2
B      -1.26815100     1.71736374     0.00000000
O      -0.05623413     1.71736374     0.00000000
```

BeF

```
2
0 2
Be     -1.39150116     1.71736374     0.00000000
F      -0.02288401     1.71736374     0.00000000
```

BeH

```
2
0 2
Be     -1.51975637     1.71736374     0.00000000
H      -0.17462876     1.71736374     0.00000000
```

CH

```
2
0 2
H      -0.22685770     1.71736374     0.00000000
C      -1.34752748     1.71736374     0.00000000
```

CO^+

```
2
1 2
C      -1.16544023     1.71736374     0.00000000
O      -0.04894493     1.71736374     0.00000000
```

Cl_2^-

```
2
-1 2
Cl     -1.84816821     1.71736374     0.00000000
Cl      0.74378302     1.71736374     0.00000000
```

OH

```
2
0 2
O      -1.21838299     1.71736374     0.00000000
H      -0.24600217     1.71736374     0.00000000
```

SH

```
2
0 2
H      -0.23623044     1.71736374     0.00000000
S      -1.57815473     1.71736374     0.00000000
```

BH_2

```
3
0 2
B       0.59641853     0.87371646     0.00000000
H       1.78009125     0.96617653     0.00000000
H      -0.07548327    -0.10514895     0.00000000
```

BeOH

```
3
0 2
Be      0.66048787     1.09741810     0.21568143
O       1.79051895     0.55448256    -0.41539753
H       2.68047417     0.79359844    -0.65004281
```

CH_2

```
3
0 3
C      -5.23402518     2.14828892    -0.02372106
H      -6.22491079     2.14828892     0.40010367
H      -4.24313961     2.14828892     0.40010367
```

CH_2^-

```
3
-1 2
C      -5.23402518     2.14828892    -0.04500367
H      -6.11026899     2.14828892     0.65395571
H      -4.35778137     2.14828892     0.65395571
```

C_2H

```
3
0 2
C       2.00432059     0.91886914    -0.00000016
C       0.79509935     0.91889280    -0.00000016
H       3.06900648     0.91889206     0.00000042
```

HCO

```
3
0 2
C     0.00000003    0.00000000   -0.00000007
H     0.00000007    0.00000000    1.11925092
O     0.97254090    0.00000000   -0.66924685
```

HCS

```
3
0 2
C       2.28173414     0.91888502     0.04077951
H       3.36929329     0.91888502     0.02762406
S       1.22188551     0.91888502    -1.10342257
```

HOO

```
3
0 2
H        3.09164018     0.91888465    -0.50669230
O        1.33015046     0.91888465    -1.11041861
O        2.23965513     0.91888465    -0.04102717
```

H_2O^+

```
3
1 2
O        1.13089528     1.42049372    -7.51430064
H        2.13230575     1.42375134    -7.51430064
H        0.79968848     2.36555222    -7.51430064
```

NH_2

```
3
0 2
H        2.13282414     1.44719198    -7.51430064
H        0.82161102     2.37386511    -7.51430064
N        1.10845441     1.38874023    -7.51430064
```

CH_3

```
4
0 2
C      -3.83793706     0.03375405     0.35029703
H      -3.32881430    -0.86459932     0.66011814
H      -3.33083677     0.75343108    -0.27190720
H      -4.85424779     0.21228141     0.66246493
```

F_2CH

```
4
0 2
C       0.08368933     0.03876414    -0.05914424
F       0.00066962     0.01888851     1.26481443
F       1.33950552    -0.11515463    -0.45866327
H      -0.43091718     0.87472301    -0.52405026
```

H_2CN

```
4
0 2
C      -3.31949068     0.14070939     1.43970841
H      -3.88586374     1.07746510     1.43961909
H      -3.88586364    -0.79604647     1.43965814
N      -2.07142492     0.14070881     1.43962247
```

NH_3^+

4

1 2

N	-0.20887250	-2.22535443	-8.03480621
H	-0.20721211	-3.24716405	-8.03508222
H	-0.20970114	-1.71469749	-7.14974932
H	-0.20970257	-1.71419768	-8.91957141

PH_3^+

4

1 2

P	-0.39815069	-2.22525203	-8.03475535
H	-0.02889662	-3.57197797	-8.03493236
H	-0.02907824	-1.55214376	-6.86829910
H	-0.02936288	-1.55204475	-9.20122309

CH_2CH

5

0 2

C	-7.33661821	0.08777664	-2.58351943
C	-6.03635541	0.26105695	-2.58350535
H	-8.02037210	0.93668453	-2.58351456
H	-7.78530204	-0.89945506	-2.58353705
H	-5.34823734	1.09205907	-2.58348863

H_2CCN

```
5
0 2
C    -0.00000006   -0.00000000    0.00000006
H    -0.00000005    0.00000004    1.07835088
H     0.92987300    0.00000004   -0.54610499
C    -1.20811030    0.00118780   -0.69171127
N    -2.22377359    0.00225813   -1.27332168
```

H_2CCO^+

```
5
1 2
C    -0.00000006    0.00000000   -0.00000020
H    -0.00000010    0.00000000    1.08460006
H     0.89904745    0.00000000   -0.60695798
C    -1.23029599    0.00000000   -0.65193578
O    -2.22576430    0.00000000   -1.17787410
```

Cartesian coordinates for large main-group compounds

Cartesian coordinates are given in Å and the xyz format with charge and multiplicity.

Aniline+

14

1 2

C	-0.25682369	-1.19856980	-0.01563094
C	1.08709089	-1.23482114	0.05770726
C	1.82006504	-0.00849652	0.13119098
C	1.12713759	1.24304328	0.12643854
C	-0.21718096	1.25432645	0.05241072
C	-0.93049194	0.04014122	-0.01962999
H	-0.82864405	-2.11350075	-0.07183026
H	1.62426935	-2.17373457	0.06219907
H	1.69434972	2.16242890	0.18247249
H	-0.75910889	2.18895233	0.04751588
H	-2.00965295	0.05922303	-0.07878513
N	3.15211890	-0.03205078	0.20423072
H	3.66409619	-0.90191060	0.20923753
H	3.69191102	0.81920808	0.25700112

4-Nitroaniline+

16

1 2

C	-0.24626433	-1.21957888	0.15717760
C	1.10000514	-1.24496155	0.13129919
C	1.83081072	-0.00958097	0.12432194
C	1.13003976	1.24288268	0.14767083
C	-0.21634057	1.24963048	0.17881545
C	-0.89416344	0.02293639	0.18203507
H	-0.84021785	-2.12181132	0.15514641
H	1.64207092	-2.18086417	0.11538350
H	1.69446164	2.16558180	0.14015233
H	-0.78798683	2.16577219	0.20553472
N	3.15500821	-0.02538262	0.09627592
H	3.67274136	-0.89283780	0.07863569
H	3.69356724	0.82946761	0.09153655
N	-2.37413965	0.04060689	0.21338432
O	-2.93745254	-1.02614708	-0.05537893
O	-2.89993255	1.12037005	0.50522071

Benzyl

14

0 2

C	-3.25470848	0.12942861	-5.20697120
C	-1.12693433	1.25554118	-5.16869592
C	-1.77148900	2.45666564	-5.07996247
C	-3.15621134	2.51201366	-5.05363077
C	-3.88790801	1.33656325	-5.11804132
H	-3.83077184	-0.78577038	-5.25699173
H	-0.04522232	1.21771104	-5.18889911
H	-1.19633704	3.37164718	-5.02991553
H	-3.66175702	3.46484768	-4.98336116
H	-4.96892912	1.37502538	-5.09779744
C	-1.84964038	0.04944050	-5.23527959
C	-1.19042867	-1.19300921	-5.32706941
H	-1.74727782	-2.11524863	-5.37796573
H	-0.11366976	-1.25066603	-5.34855580

Phenylaminyl

13

0 2

C	-0.24179056	-1.16959147	0.00825802
C	1.12164537	-1.17985460	-0.03823777
C	1.85573682	0.03405107	-0.05002704
C	1.12370582	1.24786134	-0.01199698
C	-0.23875440	1.24084159	0.03430053
C	-0.93368087	0.03570073	0.04486523
H	-0.78963448	-2.10202364	0.01675624
H	1.66577833	-2.11584390	-0.06685789
H	1.69222756	2.16684785	-0.02139728
H	-0.78462460	2.17387416	0.06294251
H	-2.01399167	0.03551737	0.08166655
N	3.19020024	0.11887586	-0.09449153
H	3.57949260	-0.82649221	-0.11794546

1,3,2-benzodithiazolyl

13

0 2

C	-4.96912354	0.00527674	0.23866063
C	-3.56618829	0.00523219	0.23853919
C	-2.85746718	1.21354213	0.23861015
C	-3.56596689	2.40981313	0.23880203
C	-4.96919112	2.40985790	0.23892295
C	-5.67776681	1.21363151	0.23885362
H	-1.77496501	1.21463430	0.23851717
H	-3.02793354	3.34839280	0.23885796
H	-5.50716462	3.34847186	0.23907180
H	-6.76026888	1.21479284	0.23894708
S	-5.66404445	-1.59925191	0.23854585
S	-2.87136999	-1.59933864	0.23830391
N	-4.26773371	-2.50384613	0.23832619

Diethylaminyl

15

0 2

C	5.34252800	0.15479186	1.02915344
C	6.86081246	0.16956300	1.03100281
H	4.94215937	1.16755975	1.02981353
H	4.97240666	-0.36384349	0.14786922
H	4.97028382	-0.36583770	1.90836482
H	7.24213975	0.71446200	0.15693630
H	7.23998552	0.71199095	1.90754375
C	8.84034029	-1.17834412	1.03033192
C	9.38295886	-2.59643098	1.03050401
H	9.20675527	-0.62523599	1.90562157
H	9.20694926	-0.62540940	0.15501226
H	10.47199108	-2.59783065	1.03090099
H	9.03517134	-3.13222490	1.91062028
H	9.03580048	-3.13220172	0.15012637
N	7.39423894	-1.17456563	1.02998901

Cyclo-hexyl

17

0 2

C	-0.80762661	-2.65112245	0.14199921
C	0.71481485	-2.74705864	0.20505608
C	1.35011595	-1.35714083	0.31900260
C	0.72452652	-0.55800661	1.41528535
C	-0.76632089	-0.53069832	1.50716385
C	-1.36267264	-1.93582483	1.37134109
H	2.42788686	-1.44050657	0.46754562
H	0.99729009	-3.34077090	1.07793570
H	1.10324699	-3.26498717	-0.67378567
H	-1.09746138	-2.09965028	-0.75786665
H	-1.24510549	-3.64754543	0.05580650
H	1.29758966	0.22682183	1.89016816
H	-1.08797609	-0.06760885	2.44134326
H	-1.17526362	0.09396129	0.69892879
H	-1.11330178	-2.51661899	2.26279912
H	-2.45150986	-1.87688851	1.32181963
H	1.21251923	-0.83844404	-0.64156926

1-adamantyl

25

0 2

C	-0.66290066	0.20234547	0.55386644
H	0.43022296	0.20312813	0.54975828
H	-1.00059750	1.24199980	0.54989692
C	-1.18387106	-0.51534588	1.82334149
H	-0.81299930	-0.00466433	2.71721182
C	-1.21664549	-0.56016370	-0.61376163
C	-0.69018625	-1.96282700	-0.69614071
H	0.40270690	-1.98032097	-0.71080390
H	-1.04741164	-2.47268484	-1.59467913
C	-0.69081068	-1.96768850	1.81277742
H	-1.03798107	-2.48309476	2.71310530
H	0.40272019	-1.99147857	1.83011200
C	-1.21131826	-2.69348071	0.56585204
H	-0.85993552	-3.72976010	0.56662257
C	-2.74484012	-2.66509501	0.56078199
H	-3.12562342	-3.18942992	-0.32055462
H	-3.12782215	-3.19268578	1.43926507
C	-2.71751054	-0.49617236	1.81296538
H	-3.07869196	0.53626177	1.83041670
H	-3.09999896	-0.98592109	2.71330406
C	-2.71339577	-0.49384747	-0.69594487
H	-3.06840168	0.53993034	-0.71045427
H	-3.08771501	-0.99128276	-1.59448783
C	-3.24663530	-1.21571530	0.56604529

```
H      -4.34078747    -1.20245745     0.56695828
```

ORCA inputs for 1-adamantyl timings

pcJ-2

```
! UHF DLPNO-CCSD ExtremeSCF RIJCOSX DLPNO-HFC2 pmodel NoFrozenCore


%pal
  nprocs 8
end


%maxcore 27000


*xyz 0 2
C      -0.66290066     0.20234547     0.55386644
H       0.43022296     0.20312813     0.54975828
H      -1.00059750     1.24199980     0.54989692
C      -1.18387106    -0.51534588     1.82334149
H      -0.81299930    -0.00466433     2.71721182
C      -1.21664549    -0.56016370    -0.61376163
C      -0.69018625    -1.96282700    -0.69614071
H       0.40270690    -1.98032097    -0.71080390
H      -1.04741164    -2.47268484    -1.59467913
C      -0.69081068    -1.96768850     1.81277742
H      -1.03798107    -2.48309476     2.71310530
H       0.40272019    -1.99147857     1.83011200
C      -1.21131826    -2.69348071     0.56585204
```

```
H      -0.85993552    -3.72976010     0.56662257
C      -2.74484012    -2.66509501     0.56078199
H      -3.12562342    -3.18942992    -0.32055462
H      -3.12782215    -3.19268578     1.43926507
C      -2.71751054    -0.49617236     1.81296538
H      -3.07869196     0.53626177     1.83041670
H      -3.09999896    -0.98592109     2.71330406
C      -2.71339577    -0.49384747    -0.69594487
H      -3.06840168     0.53993034    -0.71045427
H      -3.08771501    -0.99128276    -1.59448783
C      -3.24663530    -1.21571530     0.56604529
H      -4.34078747    -1.20245745     0.56695828
*

%basis
  Basis "pcJ-2"
  AuxJ  "cc-pVQZ/JK"
  AuxC  "cc-pwCV5Z/C"
end

%eprnmr
  Nuclei = all C {aiso}
  Nuclei = all H {aiso}
end
```

pcJ-3

```
! UHF DLPNO-CCSD ExtremeSCF RIJCOSX DLPNO-HFC2 pmodel NoFrozenCore


%pal
  nprocs 8
end


%maxcore 27000


*xyz 0 2
C      -0.66290066     0.20234547     0.55386644
H       0.43022296     0.20312813     0.54975828
H      -1.00059750     1.24199980     0.54989692
C      -1.18387106    -0.51534588     1.82334149
H      -0.81299930    -0.00466433     2.71721182
C      -1.21664549    -0.56016370    -0.61376163
C      -0.69018625    -1.96282700    -0.69614071
H       0.40270690    -1.98032097    -0.71080390
H      -1.04741164    -2.47268484    -1.59467913
C      -0.69081068    -1.96768850     1.81277742
H      -1.03798107    -2.48309476     2.71310530
H       0.40272019    -1.99147857     1.83011200
C      -1.21131826    -2.69348071     0.56585204
H      -0.85993552    -3.72976010     0.56662257
C      -2.74484012    -2.66509501     0.56078199
H      -3.12562342    -3.18942992    -0.32055462
H      -3.12782215    -3.19268578     1.43926507
```

```
C      -2.71751054    -0.49617236     1.81296538

H      -3.07869196     0.53626177     1.83041670

H      -3.09999896    -0.98592109     2.71330406

C      -2.71339577    -0.49384747    -0.69594487

H      -3.06840168     0.53993034    -0.71045427

H      -3.08771501    -0.99128276    -1.59448783

C      -3.24663530    -1.21571530     0.56604529

H      -4.34078747    -1.20245745     0.56695828

*


%basis

  Basis "pcJ-3"

  AuxJ  "cc-pVQZ/JK"

  AuxC  "cc-pwCV5Z/C"

end


%eprnmr

  Nuclei = all C {aiso}

  Nuclei = all H {aiso}

end
```
